# Evaluation of Treatment Descriptions and Alignment With Clinical Guidance of Apps for Depression on App Stores: Systematic Search and Content Analysis

**DOI:** 10.2196/14988

**Published:** 2020-11-13

**Authors:** Dionne Bowie-DaBreo, Sandra I Sünram-Lea, Corina Sas, Heather Iles-Smith

**Affiliations:** 1 Research and Innovation Centre Leeds Teaching Hospitals NHS Trust Leeds United Kingdom; 2 Department of Psychology Lancaster University Lancaster United Kingdom; 3 School of Computing and Communications Lancaster University Lancaster United Kingdom; 4 Research and Innovation Northern Care Alliance NHS Group Salford United Kingdom; 5 University of Salford Salford United Kingdom

**Keywords:** mobile mental health, mHealth, mobile apps, depression, clinical guidance, NICE guidelines, NHS, safety, ethics, content analysis

## Abstract

**Background:**

The use of apps for the treatment of depression shows great promise. However, there is uncertainty regarding the alignment of publicly available apps for depression with clinical guidance, their treatment fidelity and evidence base, and their overall safety.

**Objective:**

Built on previous analyses and reviews, this study aims to explore the treatment and safety issues of publicly available apps for depression.

**Methods:**

We conducted a content analysis of apps for depression in the 2 main UK app stores (Google Play and Apple App Store). App store listings were analyzed for intervention content, treatment fidelity, and fit with the National Institute for Health and Care Excellence (NICE) guidelines for the treatment of depression in adults.

**Results:**

A total of 353 apps for depression were included in the review. App descriptions reported the use of 20 treatment approaches and 37 treatment strategies. Many apps used transdiagnostic (155/353, 43.9%) and multitheoretical interventions to treat multiple disorders including depression. Although many interventions appeared to be evidence-informed, there were issues with treatment fidelity, research evidence, and fit with clinical guidelines. None of the apps fully aligned with the NICE guidelines for depression.

**Conclusions:**

App developers have adopted many evidence-informed treatments in their interventions; however, more work is needed to improve clinical validity, treatment fidelity, and the safety of apps. We urge developers to consult relevant guidelines and standards, and to engage in reflective questioning on treatment and safety to address these issues and to improve treatment content and intervention design.

## Introduction

### Management and Treatment of Depression

Depression is an affective disorder characterized by persistent low mood; loss of interest or pleasure; increased negative thoughts and feelings; and associated emotional, cognitive, physical, and behavioral difficulties [1-3]. Within the United Kingdom, the National Institute for Health and Care Excellence (NICE) seeks to improve outcomes for people using the National Health Service (NHS) and other public health services through the provision of evidence-based guidance, quality standards, and performance metrics. NICE guidelines for the treatment and management of depression in adults [4,5] recommend a stepped care approach of clinical and cost-effective interventions. Following early intervention through screening, assessment, and psychoeducation, first-line treatments for subthreshold or mild to moderate depression include low-intensity psychosocial interventions, specifically guided self-help based on cognitive behavioral therapy (CBT) or group physical activity programs. Persons with less severe depression who decline or do not respond well to these interventions should be offered high-intensity psychological interventions—specifically CBT, interpersonal therapy (IPT), behavioral activation, or behavioral couples therapy—or antidepressants. If declined, individuals may be offered counseling or short-term psychodynamic psychotherapy.

For moderate to severe depression, NICE advises a combination of antidepressants and high-intensity interventions (CBT or IPT), with relapse prevention consisting of antidepressants and CBT or mindfulness-based cognitive therapy (MBCT). Complex and severe cases of depression receive the highest level of care, which may include multidisciplinary care, specialist mental health services, and crisis resolution.

Network meta-analysis of clinical evidence for the treatment of depression in adults found self-help with support to be more effective than psychoeducation and self-help without support [4]. These self-help interventions included (from better to worse outcomes): computerized psychodynamic therapy with support, computerized CBT with support, computerized behavioral activation with support, computerized CBT without support, psychoeducational website, and computerized mindfulness intervention. Although the 2018 NICE draft guidelines did not specifically recommend mobile apps, their 2019 guidelines for depression in children and young people [6] advised the use of digital CBT in cases of mild depression. This included CBT delivered via a computer, tablet, or phone.

Building on this guidance, NHS England and NICE developed a digitally enabled therapy assessment program aimed at evaluating the use of digital therapy products in the NHS Improving Access to Psychological Therapies (IAPT) services [7,8]. The program assessed 14 digitally enabled therapies (ie, psychological interventions delivered on the web or through apps with the support of a therapist). Of these, 6 targeted depression in adults [9-15]. Digital therapies were assessed based on 4 criteria: content, technical standards, clinical effectiveness, and cost impact. In line with NICE guidance, content assessment of digital therapies for depression evaluated adherence to CBT and fit within a blended care model. Following expert evaluations, only 3 digital therapies were recommended for trialed use within IAPT services [11,12,14]. For those not recommended, treatment issues included misalignment with the therapist-guided model of care [10], poor user and treatment experiences [13], and incomplete treatment content for depression because of the use of a transdiagnostic approach [15]. Only one of the therapies offered a web- and app-based program [12], with others being solely web-based.

### Mobile Apps for Depression

The use of apps for the management of depression has shown promise in providing accessible and low-cost mental health interventions. Randomized controlled trials and reviews of apps for depression have reported significant reductions in depressive symptoms [16-22] and improvements in well-being [23]. There is evidence of the use of apps for assessment and psychoeducation [24], symptom tracking or mood monitoring [19,24], cognitive training and problem solving [16], and a range of treatment approaches, including CBT [19,22], behavioral therapy (BT) and dialectical behavior therapy (DBT) [22], mindfulness [19], and transdiagnostic approaches [17].

Although highlighting the potential of mobile mental health, research cautioned that findings did not reflect apps available to the public through the app marketplace. Torous et al [22] showed that only one-third of apps for depression reviewed in the literature were available for download in app stores, with research reviews of the app marketplace uncovering a worrying lack of evidence for most apps [25-27].

Content analyses and marketplace reviews of publicly available apps found hundreds of apps marketed for depression. Given the overwhelming number of apps, reviewers often limited analyses to a subset [27-30] such as apps using specific approaches [25,31,32]. The most common functionalities of apps for depression included psychoeducation [25,27,29-31], assessment [25,28,29,31], and symptom management [25,30,31]. Approximately one-third of apps for depression provided therapeutic treatment [31] or interactive interventions [29]. CBT apps for depression incorporated several strategies but were criticized for overlooking treatment processes such as challenging core beliefs and conceptualization in favor of education, monitoring and tracking, and thought records [24,30]. Overall, the authors commented that although some apps seemed to be evidence informed, reflecting some theoretical principles and strategies, the apps did not demonstrate high fidelity to evidence-based treatments such as CBT or BT [25,32] and generally lacked evidence supporting use and efficacy [21,23,25,27].

Reviews of publicly available apps for depression also highlighted insufficiencies in the treatment and safety information provided, including limited disclaimers [27], limited encouragement for users to seek in-person care [29], and inadequate reporting of affiliations or expert involvement [31,32]. Reviews of the ethics of mobile mental health [33,34] have also raised concerns with acceptance [35], risks and safety of apps [36-47], and the poverty of evidence regarding benefits and outcomes [36-40,42-46,48,49]. Therefore, there is uncertainty as to how well apps for depression match existing clinical guidelines and recommendations, their treatment fidelity (ie, adherence to components of a treatment orientation) and evidence base, and their safety for use with or without support.

### Overview of Study

This study builds on previous analyses and reviews to explore treatment descriptions of publicly available apps for depression and their alignment with clinical guidance as conveyed in app store listings. The decision to review app listings rather than downloaded apps reflects the lack of a comprehensive overview of all treatment options marketed to the public through the marketplace for apps for depression. Guided by NICE guidelines and literature on the ethics and safety of mobile mental health, we conducted content analysis of apps for depression listings in the UK app marketplace. This study aims to answer the following questions: (1) What treatment approaches and strategies are named or described in app listings of apps for depression? (2) Are treatment fidelity and evidence-informed development evident in descriptions of apps for depression? (3) Do descriptions of apps for depression reflect NICE guidelines for the treatment and management of depression? We hope that this study will advance research and discussion on the treatment content and safety of publicly available apps for depression, in particular their marketing to the public, fit with clinical guidance, and discrepancies between public health digital therapies and direct-to-consumer products. In doing so, we seek to promote improved standards and best practices in the design and marketing of mental health apps.

## Methods

### Sampling Methods

**Figure 1:**
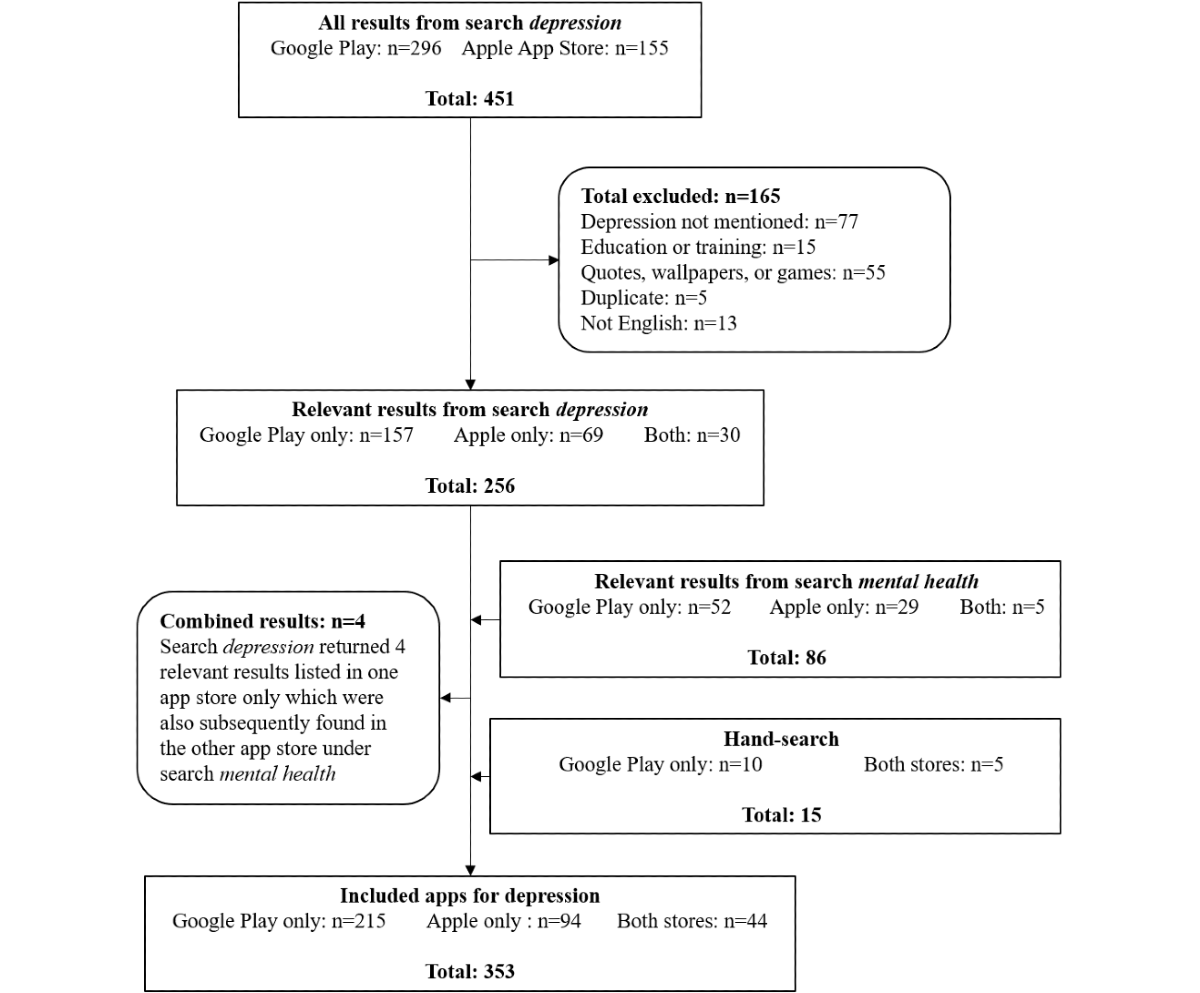
Sampling flowchart.

App search and data collection was conducted between October and November 2018, guided by methods used by Shen et al [31] and Stawarz et al [32]. The first step involved search of apps in the 2 main UK app stores: Google Play and Apple App Store. The initial search was performed using the search term *depression*. For the review, apps for depression were defined as apps with app store listings mentioning depression or depressive symptoms. Apps were included in the review if they met the following criteria: (1) app description included terms *depression*, *low mood* or *mood disorder*, *mood management*, *negative thoughts*, or *distress* and (2) app listing was in English. Apps were excluded if they (1) did not mention depression or depressive symptoms, (2) were for professional training, (3) only provided quotes or wallpapers, or (4) were duplicates, that is, copies of an app listed within the same app store. Duplicates did not include free and paid versions of apps or apps listed in both stores; in these cases, all relevant apps were included in the review. Apps were also not excluded if they targeted another mental health problem (eg, anxiety) once they mentioned depression or depressive symptoms. This initial search returned 451 apps (296 in Google Play and 155 in Apple App Store). Of these, 256 unique apps met the inclusion criteria (Figure 1).

A second search of the same app stores was performed using the term *mental health* aimed at detecting apps for depression that were not returned in the primary search. Finally, a hand search of the same app stores for apps for depression that were reported in previous research but not returned in the searches was performed. These searches yielded an additional 97 eligible apps. This resulted in a total of 353 unique apps for depression being included in the analysis (Multimedia Appendix 1).

### Content Analysis

This study aims to explore treatment descriptions and fit with clinical guidance of apps for depression, as evident in app store listings and websites. Before the review, a list of variables was compiled to extract data on app information, developer information, treatment information, and usage. Data were initially extracted from app listings and websites verbatim or using yes or no coding to indicate the presence or absence of variables. Throughout this process, coding was developed iteratively as treatment information emerged. Treatment codes were informed by NICE guidelines [4-6], literature on the treatment of depression [50,51], and app reviews [25,29]. Final coding is presented in Multimedia Appendix 2. Data extraction and coding were led by the first author and revised through group discussion until consensus was reached among all authors.

Descriptive statistics of the app data were computed using SPSS version 25. Categorical variables were recoded numerically before analysis. Spearman rank correlation coefficient was used to explore associations within- and between-treatment approaches and treatment strategies as part of the analysis of treatment fidelity and evidence-informed interventions. Chi-square tests were also performed to determine associations between developer type and treatment variables.

## Results

### Treatment Descriptions of Apps for Depression

App store descriptions typically touted the suitability and benefits of apps for depression or related difficulties. The findings indicate that 28.3% (100/353) of apps targeted solely depression, with most apps targeting depression alongside other difficulties, notably anxiety and stress (Table 1). Just under one-half (174/353, 49.3%) of all apps targeted multiple (4 or more) disorders.

Less than one-third of apps (108/353, 30.6%) offered a disorder-specific intervention, that is, an intervention designed to treat a single mental health problem (eg, depression), whereas 43.9% (155/353) of apps described transdiagnostic interventions treating multiple disorders using the same treatment content. A further 25.5% (90/353) of apps reported treatment of multiple disorders with varied content for each.

In this analysis, the treatment approach was defined as theoretical or treatment orientation such as CBT, whereas treatment strategies were the techniques employed in the delivery of the intervention, such as cognitive reappraisal. Our review identified 20 treatment approaches and 37 treatment strategies. For some apps, approaches (36/353, 10.2%) and strategies (115/353, 32.6%) were not clearly presented. As per previous research, the most common approach was psychoeducation, with assessment and CBT frequently used (Table 2). There was also a high use of complementary and alternative therapies.

**Table 1:** Frequency of target disorders in apps for depression (N=353).

Target disorders	Apps, n (%)
Multiple	174 (49.3)
Depression	100 (28.3)
Depression and anxiety	34 (9.6)
Depression, anxiety, and stress	27 (7.7)
Suicide or self-injury	4 (1.1)
Anxiety and stress	3 (0.8)
Stress	3 (0.8)
Depression, anxiety, and bipolar disorder	2 (0.6)
Anxiety	1 (0.3)
Sleep	1 (0.3)
Depression and stress	1 (0.3)
Depression and bipolar disorder	1 (0.3)
Depression, bipolar disorder, and schizophrenia	1 (0.3)
Depression, anxiety, and trauma	1 (0.3)

**Table 2:** Treatment approaches of apps for depression (N=353).

Treatment approach	Apps, n (%)^a^
Psychoeducation	141 (39.9)
Complementary and alternative therapies	79 (22.4)
Screening or assessment	66 (18.7)
Cognitive behavioral therapy	49 (13.9)
Psychosocial	46 (13.0)
Self-help	33 (9.4)
Online therapy	19 (5.4)
Positive psychology	13 (3.7)
Exercise	12 (3.4)
Dialectical behavior therapy	5 (1.4)
Acceptance and commitment therapy	4 (1.1)
Cognitive training	4 (1.1)
Spiritual or faith based	3 (0.9)
Behavioral therapy	2 (0.6)
Eye movement desensitization and reprocessing	2 (0.6)
Interpersonal therapy	1 (0.3)
Mindfulness-based cognitive therapy	1 (0.3)
Motivational interviewing	1 (0.3)
Neurostimulation	1 (0.3)
Problem-solving therapy	1 (0.3)

^a^Percentages do not add up to 100% because some apps use multiple approaches.

**Figure 2:**
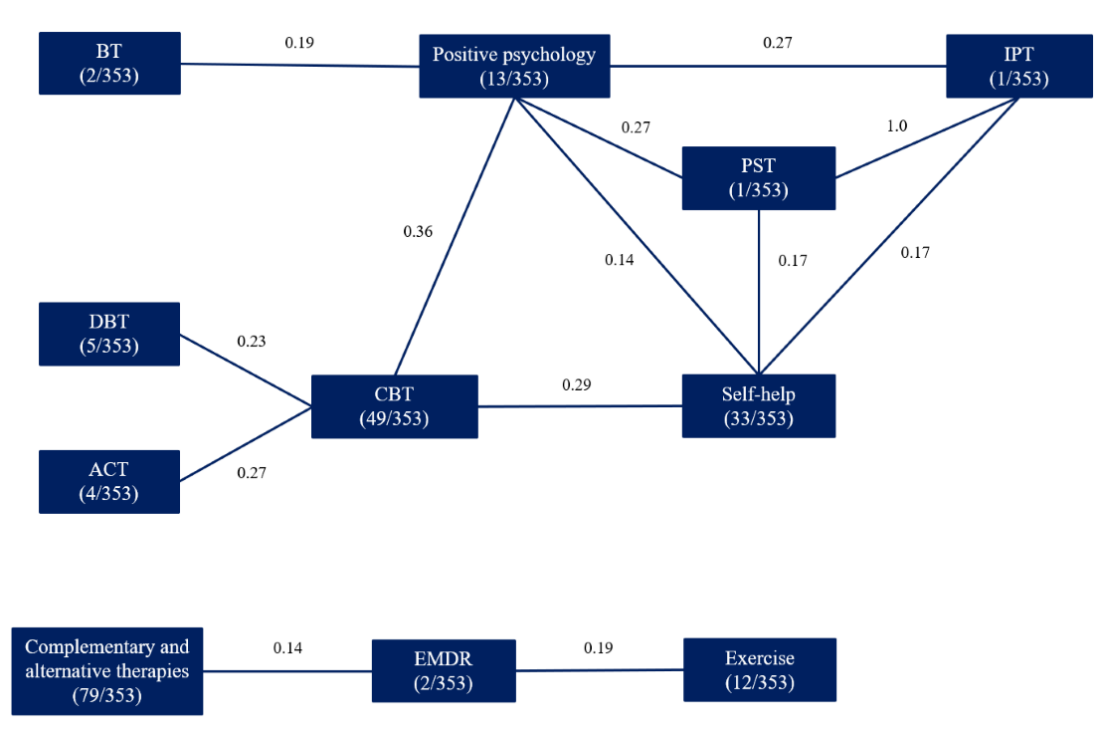
Significant associations between treatment approaches in apps for depression (Spearman rank correlation coefficients, *P*<.01). ACT: acceptance and commitment therapy; BT: behavioral therapy; CBT: cognitive behavioral therapy; DBT: dialectical behavioral therapy; EMDR: eye movement desensitization and reprocessing; IPT: interpersonal therapy; PST: problem-solving therapy.

Our review found that 59.2% (209/353) of app listings reported only 1 identifiable treatment approach, with others describing combinations of 2 to 6 approaches (n=317 [missing cases excluded]; mean 1.53, SD 0.92; mode 1). There were 59 unique combinations of approaches. This is captured in Figure 2, which maps significant positive associations between treatment approaches, providing insight into the most commonly used treatment combinations (the correlation table is given in Multimedia Appendix 3). Despite the low frequency of some approaches, these results illustrate patterns in the treatments used, such as combinations of different cognitive approaches (acceptance and commitment therapy, CBT, and DBT).

Eclecticism in treatment was also evident in the variety of treatment strategies (Table 3). Overall, 22.9% (81/353) of apps described only 1 identifiable treatment strategy, with the remaining naming between 2 and 16 strategies (n=238 [missing cases excluded]; mean 2.53, SD 1.82; mode 1). There were 112 unique combinations of strategies (Multimedia Appendix 1).

**Figure 3:**
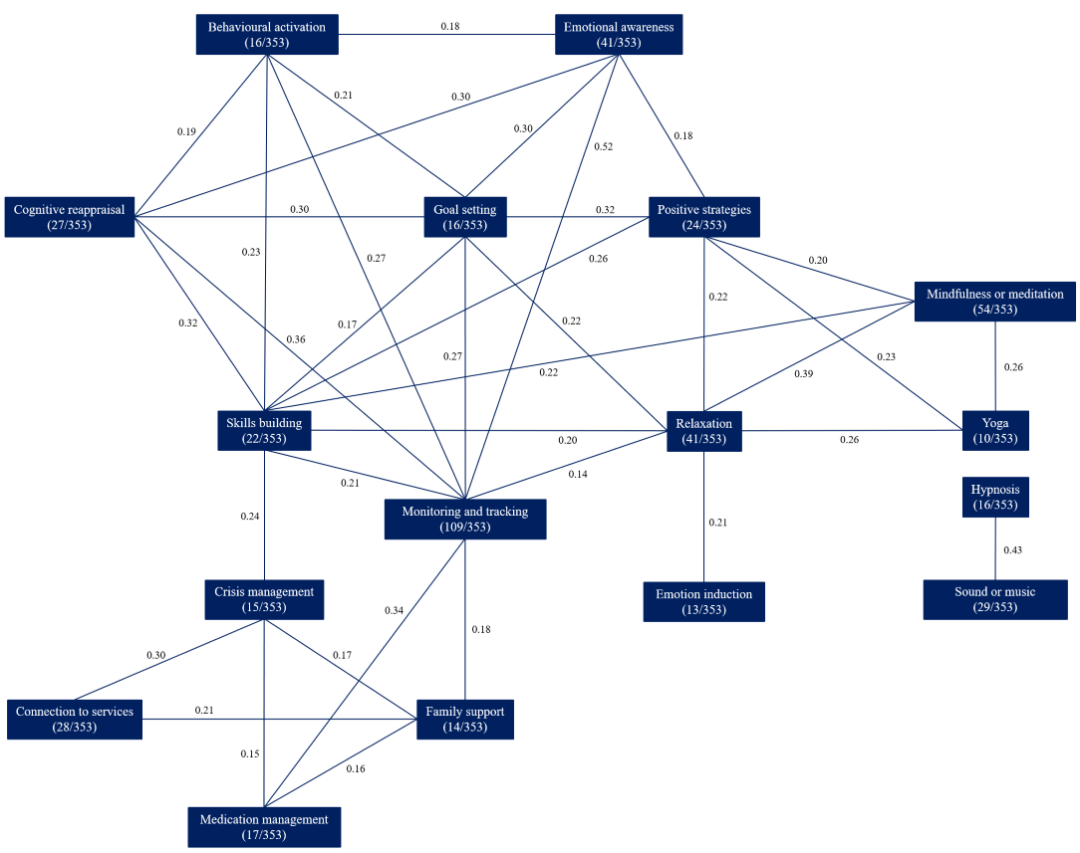
Significant associations between most common treatment strategies in apps for depression (Spearman rank correlation coefficients, *P*<.01).

These strategy combinations are illustrated in Figure 3, which captures the significant positive associations between the most commonly identified strategies (n>9; the correlation table is given in Multimedia Appendix 4). As with the treatment approaches, patterns emerged in the reported use of treatment strategies. Such patterns suggest evidence-informed development, as seen with the associations between the use of emotional awareness, cognitive reappraisal, behavioral activation, and monitoring and tracking, which are all emotion regulation techniques typically employed in treatments for depression, such as CBT and evidence-based multitheoretical [50] and transdiagnostic [51] approaches.

**Table 3:** Treatment strategies of apps for depression (N=353).

Treatment strategies	Apps, n (%)^a^
Monitoring and tracking (including diaries)	109 (30.9)
Mindfulness or meditation	54 (15.3)
Emotional awareness	41 (11.6)
Relaxation	41 (11.6)
Peer support	34 (9.6)
Sound or music	29 (8.2)
Connection to services	28 (7.9)
Cognitive reappraisal	27 (7.7)
Positive strategies	24 (6.8)
Lifestyle or nutrition	22 (6.2)
Skills building	22 (6.2)
Medication management	17 (4.8)
Behavioral activation	16 (4.5)
Goal setting	16 (4.5)
Hypnosis	16 (4.5)
Crisis management	15 (4.2)
Family support	14 (4.0)
Emotion induction	13 (3.7)
Yoga	10 (2.8)
Distraction or grounding	5 (1.4)
Acupressure	4 (1.1)
Chatbot	4 (1.1)
Self-compassion	4 (1.1)
Bodily awareness	3 (0.9)
Coaching	3 (0.9)
Gamification	3 (0.9)
Motivation enhancement	3 (0.9)
Problem solving	3 (0.9)
Cognitive bias modification	2 (0.6)
Exposure	2 (0.6)
Neuro-linguistic programming	2 (0.6)
Acceptance	1 (0.3)
Art therapy	1 (0.3)
Chromotherapy	1 (0.3)
Emotional freedom techniques	1 (0.3)
Havening	1 (0.3)
Transcranial direct current stimulation	1 (0.3)

^a^Percentages do not add up to 100% as some apps use multiple strategies.

CBT was associated with 13 of the 37 strategies (where significant associations are *P*<.01), including cognitive reappraisal, monitoring and tracking, and emotional awareness (Table 4).

Mindfulness meditation was also often used in CBT apps; however, only one app was identified as having a mindfulness-based cognitive approach. Although these associations suggested some evidence-informed development, there were shortcomings in the reported use of these strategies across CBT app listings. Specifically, except for monitoring and tracking (41/49, 84%), less than half of all CBT app store descriptions mentioned the use of these strategies (Multimedia Appendix 5). Only 49% (24/49) CBT app listings described the use of cognitive reappraisal, whereas 45% (22/49) mentioned emotional awareness, 16% (8/49) used goal setting, and 14% (7/49) reported the use of behavioral activation. In addition, although a high number of CBT apps employed the use of monitoring and tracking of mood, thoughts, and behaviors, fewer reported the use of screening or assessment (8/49, 16%), with only half of these app listings naming the measure used. More often, CBT apps described the use of psychoeducation (15/49, 31%), although this too was underutilized or underreported.

**Table 4:** Spearman rank correlation coefficients for cognitive behavioral therapy and all strategies (N=353).

Treatment strategies	Spearman correlation coefficient, ρ	*P* value
Cognitive reappraisal	0.62	<.001
Monitoring and tracking (including diaries)	0.44	<.001
Emotional awareness	0.42	<.001
Skills building	0.30	<.001
Relaxation	0.29	<.001
Goal setting	0.23	<.001
Mindfulness or meditation	0.22	<.001
Behavioral activation	0.19	<.001
Chatbot	0.19	<.001
Exposure	0.19	<.001
Self-compassion	0.19	<.001
Positive strategies	0.15	.004
Coaching	0.14	.008
Emotional freedom techniques	0.13	.01
Havening	0.13	.01
Connection to services	–0.12	.03
Crisis management	0.12	.03
Sound or music	–0.09	.09
Cognitive bias modification	0.08	.14
Neuro-linguistic programming	0.08	.14
Yoga	0.08	.14
Bodily awareness	0.05	.33
Motivation enhancement	0.05	.33
Problem solving	0.05	.33
Hypnosis	–0.05	.37
Family support	0.04	.41
Acupressure	–0.04	.42
Gamification	–0.04	.49
Distraction or grounding	0.02	.69
Acceptance	–0.02	.70
Art therapy	–0.02	.70
Chromotherapy	–0.02	.70
Transcranial direct current stimulation	–0.02	.70
Medication management	–0.01	.80
Emotion induction	0.01	.87
Peer support	0.008	.88
Lifestyle or nutrition	–0.002	.97

### Alignment With Clinical Guidelines on the Treatment of Depression

In terms of adherence to clinical guidance, 67.1% (237/353) of apps reported the use of at least one treatment approach recommended in NICE guidelines for the treatment of depression. Over half of all apps (181/353, 51.3%) described an early intervention, namely, assessment or psychoeducation, whereas 19.8% (70/353) of apps described the use of a NICE-recommended psychological approach (ie, CBT, BT, IPT, or web-based therapy).

Although 13.9% (49/353) of apps adhered, to some extent, to NICE’s recommendation for digital CBT, only 3.7% (13/353) of CBT apps suggested use with in-person support (Multimedia Appendix 5). Most CBT apps (37/49, 76%) appeared to use a transdiagnostic approach to treat multiple disorders, including depression. However, app descriptions did not specifically address the treatment of comorbidities or the suitability for use in complex cases. Moreover, only 18% (9/49) of CBT apps appeared to have published research on use or outcomes. In total, only 1 CBT app (MoodKit-Mood Improvement Tools) was found to have both published research and advice to use on its own or to enhance professional treatment. However, this app was not marketed as a comprehensive CBT program but rather as a mood improvement toolbox incorporating principles and techniques of CBT. Overall, none of the app store descriptions aligned with clinical guidance when assessed for evidence of NICE-recommended evidence-based interventions, therapist-guided models of care, and clinical effectiveness.

### Further Treatment and Safety Issues

Overall, evidence of app use, safety, and outcomes was not available for most apps (314/353, 89.0%). Despite this, most app store descriptions (285/353, 80.7%) did not provide a disclaimer regarding treatment, appropriate use, or limitations. When provided, disclaimers ranged from caution that the app does not replace traditional care, guidance to contact a health care provider in cases of emergency, explicit statements of when the app should not be used, or nonliability claims. Less common but concerning were instances where app descriptions included inaccurate information (15/352, 4.3%)—such as unsupported claims that specific techniques (eg, daily journaling) were the most effective in treating depression—or unsafe claims (8/352, 2.3%), for instance, unsupported statements that users would not need to see a health care professional, would not experience any risks or harms, or would experience immediate benefits.

Our review of the identified skills and expertise of developers found that about one-third (117/353, 33.1%) of apps for depression explicitly mentioned the involvement of health care professionals, either in consultation or as a part of the development team. As many as 57.8% (204/353) of apps appeared to be developed by private entities without explicit mention of the involvement of health care or other multisector stakeholders. The importance of multidisciplinary development teams was reflected in the absence of research conducted by private entities without mention of stakeholder involvement—specifically, all but one app, which reported published (29/353, 8.2%) or unpublished (10/353, 2.8%) research involved health care (22/39, 56%) or academia (17/39, 44%). Differences in developer type and treatment approach were also noted. The reported use of psychoeducation was associated with development by private entities without stakeholders (χ²_1_=5.4; *P*=.02) but was less associated with academia (χ²_1_=4.5; *P*=.04). Private entities (without stakeholders) were also associated with the use of complementary and alternative therapies (χ²_1_=4.7; *P*=.03) but less associated with the use of CBT (χ²_1_=14.7; *P*<.001). Comparatively, development teams with health care were associated with the use of CBT (χ²_1_=20.3; *P*<.001), with 61% (30/49) of CBT apps explicitly mentioning the involvement of health care in app design or development.

## Discussion

### Principal Findings

The app marketplace offers potential users a range of apps marketed for the treatment and management of depression. To the best of our knowledge, this study is the first to provide a comprehensive review of treatment descriptions of publicly available apps for depression, exploring all reported treatment approaches and strategies and the interrelations between them. In doing so, we considered issues of treatment fidelity and the quality of information presented to potential users to allow them to make informed treatment decisions. This research is particularly novel in its consideration of the alignment of publicly available apps for depression with clinical guidelines [4,5]. Our findings highlighted notable shortcomings in treatment descriptions and clinical relevance, demonstrating the need for improved regulation and evaluation of direct-to-consumer mental health technologies.

### Treatment Descriptions of Apps for Depression

#### The Popularity of Transdiagnostic Approaches

App store descriptions provided a range of treatment information, with no standardized reporting of intervention details, such as target disorder, intervention type, and treatment approaches and strategies. As such, there was wide variation in the amount and quality of treatment information provided by different apps.

With regard to target disorder, less than one-third of apps targeted solely depression, with the majority marketed for multiple disorders. To cater to this multiplicity of mental health problems, over 40% (155/353, 43.9%) of apps adopted transdiagnostic approaches. Proponents of transdiagnostic approaches [51-54] highlight the shared constructs and mechanisms underlying many disorders, suggesting benefits in the development and use of treatments across multiple mental health problems. Sauer-Zavala et al [54] presented 3 categories of transdiagnostic approaches, namely, universally applied therapeutic principles, as seen in the application of CBT to treat multiple disorders; modular treatments, whereby evidence-based strategies are selected based on a client’s individual needs rather than diagnosis; and shared mechanism treatment that targets the underlying mechanisms in a class of disorders. There is potential value in the development of transdiagnostic apps for the treatment of depression [17]. However, as seen in the categorizations of transdiagnostic approaches, such treatments require a strong evidence base and rationale underlying development and use. Developers seeking to design transdiagnostic interventions should therefore consider the type of transdiagnostic approach to be employed, the evidence base underlying their intervention, and the evidence needed to justify use and effectiveness with the target populations. Although many apps appeared to adopt transdiagnostic approaches, this was not explicitly stated in app listings, with none reporting use of existing transdiagnostic treatment models [51,53].

#### Treatment Approach and Evidence-Informed Development

Developers typically described at least one treatment approach, with several app descriptions reporting the use of 2 or more approaches. Psychoeducation was the most popular approach, as per previous reviews [25,27,29-31]. This is not surprising given the relative ease of creating informational apps rather than interactive interventions. More surprising was the frequency of complementary and alternative approaches, which were more common than assessment and CBT. This may also reflect the ease in developing complementary and alternative app therapies, which typically provided content such as sound, music, or hypnosis or mindfulness meditation recordings. This was supported by our findings that private entities without stakeholder input were more likely to develop psychoeducation and complementary and alternative app therapies rather than interactive evidence-based interventions. Although complementary and alternative therapies may offer supplementary management of mental health difficulties, research is needed to demarcate how and by whom such apps should be used, the effectiveness and outcomes of their use for depression, and fit within existing evidence-based treatment models.

Our analysis also identified 37 unique treatment strategies, with just under half of all apps describing the use of 2 or more strategies. The plethora of strategy combinations suggested idiosyncrasies in depression treatments or their marketing within app stores. Without standardized reporting of treatment approaches and strategies in app stores, potential users are left to decipher app store descriptions for clues of the intervention before download. Further research is therefore needed to determine how accurately app interventions reflect their app store descriptions and the range of unique combinations of approaches and strategies in depression treatments as determined by use of the apps.

This study provides novel insights into patterns of approaches and strategies across all apps for depression, with significant associations found between theoretically similar orientations and methods. CBT apps for depression demonstrated significant associations with several elements of CBT, including cognitive reappraisal, monitoring and tracking, goal setting, behavioral activation, emotional awareness, and skills building [25,55-57]. However, except for monitoring and tracking, less than half of all CBT apps for depression reported use of these strategies. Moreover, as noted by Huguet et al [25], other core elements were either underreported or absent. Therefore, apps demonstrated some treatment fidelity in their described interventions; however, there remained significant gaps in descriptions of theoretical principles and methods.

#### Eclecticism in Apps for Depression

Although many apps appeared to lack the core elements of specific approaches, several apps reported the use of multiple approaches and unique combinations of strategies. These seemingly multitheoretical interventions are reflective of the eclecticism in treatment seen in traditional mental health care [58-61]. In real-world settings, decisions to offer eclectic or integrative treatments require clinician judgment, knowledge, and expertise to adapt established interventions to meet the needs of individual cases. In this sense, integrative care may be more complex than manualized treatments; therefore, they may be more difficult to deliver effectively outside of in-person care. Most mental health apps do not benefit from flexible clinical decision making and in-the-moment expertise and thus require clear information and evidence supporting their methods, use, and outcomes. Therefore, the lack of research for most apps is concerning and questions the validity of apps to deliver the promised effects. Apps did not report use of multitheoretical treatment models [50,58] but rather tended to cite benefits of individual treatment approaches that were then combined in their intervention. This design approach further supported the suggestion that apps were evidence informed rather than evidence based [25,27,32].

### Alignment With Clinical Guidelines on the Treatment of Depression

Another important consideration in assessing app validity and suitability is their alignment with clinical guidance. For our study, we focused on fit with NICE guidelines for the treatment and management of depression in adults [4,5,7]. First, we considered the reported use of NICE-recommended treatment approaches that are not specific to mobile mental health. We then considered alignment with NICE recommendations on digital therapies for depression, specifically the use of CBT, provisions for in-person support, and evidence of clinical effectiveness. Superficially, approximately two-thirds of app descriptions mentioned the use of treatment approaches outlined in NICE guidelines, that is, at face value, most apps appeared to incorporate aspects of clinically recommended treatments, including psychoeducation, CBT, IPT, and BT [4,5,50,62,63]. A more detailed review of these apps is warranted to determine fidelity to these approaches.

Specific reference to the use of mobile mental health within NICE guidelines was reflected in recommendations for guided self-help [4] and digital CBT [6,7]. Few apps fit this framework, with only 13 apps offering CBT with suggested use with practitioner support. Given the lack of evidence of clinical effectiveness of these apps and pervasive shortcomings in descriptions of core components of CBT, we did not find any of the 353 app listings reviewed to fully align with treatment criteria in NICE guidelines. In their present state, apps may be more suited to provide supplement treatment for depression through their focus on specific aspects of care, such as mood tracking or goal setting. There were several strategies in apps for depression that aligned with aspects of treatment in the NICE framework, namely, apps offering monitoring and tracking, mindfulness, peer support, crisis management, and medication management. These strategies could act as tools to support treatment within the stepped care model utilized by the NHS. For example, monitoring and tracking apps could be utilized in active monitoring, whereas medication management apps could play an important role in increasing patient adherence to pharmacological treatments [64,65]. NICE analysis of the clinical effectiveness of mindfulness and peer support found both offered potential benefits in treating and managing depression but lacked enough evidence to support recommended use [4]. Therefore, there is some justification in the use of these techniques to complement clinically effective treatments, as determined by individual needs and preferences of users.

### Implications for App Design, Development, and Marketing

This study highlights notable shortcomings in the treatment design and marketing of direct-to-consumer apps for depression. There are persisting uncertainties regarding the treatment fidelity and validity of these apps, with more research needed to support the prevalent use of transdiagnostic and multitheoretical approaches and complementary and alternative app therapies. Our review shows a marked discrepancy between the digital interventions recommended for use within the UK public health system and apps for depression available to the public via the app marketplace. Developers hoping to create digital therapy products for use within the NHS should consult relevant guidance [4-7,63,66], standards [67-70], and technical specifications [71,72] to ensure that their app aligns with key criteria, including the use of evidence-based treatments, provisions for blended care, and evidence of clinical effectiveness. Although developers not targeting the public health system are not bound by these guidelines, we urge all developers of apps for depression to be au fait with best practices and to use these as a foundation in developing their digital interventions and innovations. New treatment approaches and methods are encouraged but must emerge from existing knowledge, evidence, and best practices.

In creating and distributing mental health interventions, app developers have a duty of care and responsibility for the content they design and develop and how it is marketed to the public. Regardless of the choice of treatment, developers and app stores have an obligation to provide potential users with enough information to help them make informed decisions regarding an app’s suitability for their needs. Insufficient treatment information and lack of research evidence impair the abilities of potential users to make safe and informed choices and to adequately prepare for risks and outcomes [44,73]. The lack of evidence-based apps and the eclectic nature of some interventions warrant greater safety considerations given the use by potentially vulnerable persons. Efforts should be made to assess and mitigate potential risks and harms, to protect vulnerable groups, and to provide potential users with accurate and transparent information regarding treatment, safety, and outcomes. App stores should facilitate standardized reporting of information about target users, target disorders, intervention type, treatment approach, clinical evidence, compliance with guidelines and standards, expected benefits and outcomes, potential risks, safety and safeguarding, and general guidance on use and stage in treatment. App listings should also clearly outline the level of support provided and guidance on additional support for optimal use and outcomes.

Developers are encouraged to embrace the complexities of mobile mental health and to be innovative in their intervention design and development through multidisciplinary collaboration to produce clinically valid, effective, and safe treatments. To facilitate this process, we present several reflective questions for developers to engage with at the outset and throughout the design and development of mental health technologies. These questions aim to help developers to frame the rationale for their intervention, to assess their strengths and limitations in the design and development process, and to consider the treatment and safety needs involved.

### Reflective Questioning for the Design and Development of Mobile Mental Health

#### Skills and Expertise

The following questions encourage reflection on the skills and expertise of the development team, specifically their competencies, multidisciplinary expertise, and user involvement:

- What knowledge and skills do you require for your project to be a success?
- Are you part of a multidisciplinary team? Do you need expert involvement?
- Is the development team sufficiently knowledgeable and skilled in clinical care, psychological interventions, theoretical principles, evidence-based practices, safety and safeguarding, clinical guidelines and standards, ethics, and codes of conduct?
- How will you involve user groups in planning, design, development, and research?

#### Treatment Design

Questions on treatment design aim to assist developers in considering the rationale for their app interventions, the intended target disorders and users, and the appropriate treatment orientation and strategies required to produce a quality app:

- Who are you developing the app for (consider demographics, target disorder, mental health difficulties and needs of users, and treatment histories and needs of users)?
- Why have you selected that group of target users?
- What stage of treatment will your app address (consider early intervention, first-line intervention, relapse prevention, crisis management, etc)?
- Will your app span more than one treatment stage? How will this be achieved through your intervention and app design?
- Do you intend your app to be used as a standalone intervention or supplementary to traditional care? How will this be reflected in your intervention and app design?
- What level of support will you provide users (consider connection to in-person services, crisis management, blended care, etc)?
- Do you wish to develop a disorder-specific intervention, or will you target multiple disorders using a transdiagnostic approach?
- What type of transdiagnostic approach will you employ (consider the 3 categories of transdiagnostic approaches discussed by Sauer-Zavala et al [54])?
- Will your app intervention target the disorder (eg, depression) or specific symptoms (eg, poor sleep)? How will this be reflected in your intervention and app design?
- What is your treatment approach?
- Why have you chosen this treatment approach? How does it align with your rationale, skills and expertise, and intervention design?
- How does your app intervention reflect evidence-based treatments and clinical guidelines?
- For app interventions adapted from evidence-based treatments (eg, CBT), will your app include all core treatment elements? How will this be achieved through your intervention and app design?
- What innovations will your app offer? Will these fit the existing models of care?
- What would make this project a success for you?

#### Safety and Duty of Care

Developers should also reflect on the safety of mental health apps and their role in designing, developing, and maintaining safe mobile mental health:

- What do you consider to be your roles, responsibilities, and duty of care as the developer of a mental health app? How is this reflected in your app design, development, and marketing?
- What values are important to you in the design, development, and deployment of this technology?
- Has your app been designed, developed, and marketed with safety in mind?

We encourage developers to use these reflections not only throughout design and development but also in the deployment, maintenance, and marketing of their apps.

### Limitations

This study explores treatment descriptions and alignment with the clinical guidance of apps in the UK app marketplace for depression. It builds on previous content analyses to provide a comprehensive overview of treatment content and clinical validity as evident in app store descriptions; however, it is not without limitations. First, we chose to conduct manual search and data extraction of app listings rather than to use a script to pull data from the app stores. Both methods have been used in previous marketplace reviews and offer their own benefits and limitations. Our decision to perform manual search reflects our focus on the marketing of apps to the public, with this method allowing for a first-person experience of searching, identifying, and evaluating all returned apps. This meant that search results—and therefore the final list of apps reviewed—may differ slightly from those returned through a script. However, we believe our findings benefited from the firsthand navigation of the app marketplace and the challenges potential users may encounter in their search for suitable apps.

Similarly, although a strength of the review was the inclusion of all apps for depression, this limited our focus on app store descriptions rather than downloaded apps. Our review captured issues in the marketing and treatment descriptions of apps but acknowledges that there may be discrepancies between app store listings and in-app content. Therefore, there is scope for further research to explore these issues through the use and in-depth evaluation of apps. The iterative nature of the review also allowed for rich data collection but limited rigorous research methods such as blinded coding and interrater reliability. Therefore, future reviews would benefit from the use of these methods to strengthen the current findings.

Although the analysis was largely descriptive, correlation calculations were performed to explore relationships within- and between treatment approaches and strategies. The large number of calculations may have resulted in increased type II errors. To mitigate this, findings are reported as significant at *P*<.01. We opted to not perform a correction calculation (eg, the Bonferroni correction or false discovery rate), as we believe it more beneficial for the development of future research to limit the risk of type I errors, which would exclude potentially interesting findings from the discussion. Results should be interpreted with this in mind. Finally, this study was framed and conducted within the United Kingdom. It is expected that findings will be relevant to other health care markets; however, country-specific practices may exist. The application of findings should be done with this in mind.

### Conclusions

This study advanced previous content analyses by providing a comprehensive overview of treatment descriptions of publicly available apps for depression. This is the first content analysis of apps for depression to explore the full range of reported treatment approaches and strategies and their fit with clinical guidelines. App developers have adopted many evidence-based treatments in their interventions; however, much work remains in improving the validity, fidelity, clinical relevance, and safety of apps offered directly to consumers. We encourage developers to consult guidelines and standards and engage in reflective questioning regarding treatment and safety. Developers are urged to transfer this information to potential users through transparent and sufficiently detailed app listings to allow users to make informed decisions before app download and use.

## Appendix

Multimedia Appendix 1 List of all 353 apps for depression included in the review.

Multimedia Appendix 2 Full code list used in content analysis of apps for depression.

Multimedia Appendix 3 Spearman rank correlation coefficients for treatment approaches.

Multimedia Appendix 4 Spearman rank correlation coefficients for treatment strategies.

Multimedia Appendix 5 Treatment content of cognitive behavioral therapy apps for depression.

## Abbreviations

BT: behavioral therapy
CBT: cognitive behavioral therapy
DBT: dialectical behavioral therapy
IAPT: Improving Access to Psychological Therapies
IPT: interpersonal therapy
MBCT: mindfulness-based cognitive therapy
NHS: National Health Service
NICE: National Institute for Health and Care Excellence
