# Supplementary material for: Evaluation of Treatment Descriptions and Alignment With Clinical Guidance of Apps for Depression on App Stores: Systematic Search and Content Analysis
Source: JMIR Form Res. 2020 Nov 13;4(11):e14988. doi: 10.2196/14988 (PMC7695532; doi:10.2196/14988)
Supplement: Multimedia Appendix 2 [file formative_v4i11e14988_app2.docx]

Full code list used in review and content analysis of apps for depression

| Variable | Code | Description |
| --- | --- | --- |
|  |  |  |
| **App name** | Recorded verbatim | Full name of app as listed in app store |
| **Developer name** | Recorded verbatim | Name of developer as listed in app store |
| **Developer type** |  |  |
|  | Academic | App store listing or website explicitly stated involvement of academia in app development |
|  | Health care | App store listing or website explicitly stated involvement of health care professional or agency in development |
|  | Government | App store listing or website explicitly stated involvement of government or state agency in app development |
|  | NGO | App store listing or website explicitly stated involvement of non–governmental organization in app development |
| **Target disorder** | Recorded verbatim | All target disorders named in app listing |
| **Treatment approach** |  |  |
|  | ACT | App explicitly described as using acceptance and commitment therapy (ACT) |
|  | Screening or assessment | App described as providing screening or assessment, with or without mention of specific assessment tools |
|  | BT | App explicitly described as using behavioral therapy (BT) |
|  | Complementary and alternative therapies | App described as using complementary or alternative therapies through naming of associated strategies and therapies |
|  | CBT | App explicitly described as using cognitive behavioral therapy (CBT) |
|  | Cognitive training | App described as using cognitive training or associated approaches |
|  | DBT | App explicitly described as using dialectical behavioral therapy (DBT) |
|  | EMDR | App explicitly described as using eye movement desensitization and reprocessing (EMDR) |
|  | Exercise | App described as using exercise or physical activity |
|  | IPT | App explicitly described as using interpersonal therapy (IPT) |
|  | MBCT | App explicitly described as using mindfulness–based cognitive therapy (MBCT) |
|  | MI | App explicitly described as using motivation interviewing (MI) |
|  | Neurostimulation | App described as using neurostimulation or associated approaches |
|  | Online therapy | App described as providing in–app on–demand healthcare services |
|  | Positive psychology | App explicitly described as using positive psychology or its principles |
|  | PST | App explicitly described as using problem–solving therapy (PST) |
|  | Psychoeducation | App described as providing psychoeducational content |
|  | Psychosocial | App described as using psychosocial treatments or associated approaches |
|  | Spiritual or faith–based | App described as using spiritual or faith–based approaches |
|  | Self–help | App explicitly described as self–help |
| **Treatment strategy** |  |  |
|  | Acceptance | App explicitly described as using acceptance as a treatment strategy |
|  | Acupressure | App explicitly described as using acupressure |
|  | Art therapy | App described as using art or art therapy |
|  | Behavioral activation | App described as using behavioral activation or associated strategies |
|  | Bodily awareness | App described as using strategies focused on bodily or physical awareness |
|  | Chatbot | App described as using a chatbot or AI |
|  | Chromotherapy | App explicitly described as using chromotherapy |
|  | Coaching | App explicitly described as using coaching |
|  | Cognitive bias modification | App described as using cognitive bias modification or associated strategies |
|  | Cognitive reappraisal | App described as using cognitive reappraisal or associated strategies |
|  | Connection to services | App described as providing connection to services including links to real–world help and support |
|  | Crisis management | App described as providing crisis management including crisis plans |
|  | Distraction or grounding | App described as using distraction, grounding, or associated strategies |
|  | EFT | App explicitly described as using emotional freedom techniques |
|  | Emotional awareness | App described as using strategies focused on emotional awareness |
|  | Emotion induction | App described as using emotion induction or strategies to alter mood |
|  | Exposure | App explicitly described as using exposure |
|  | Family support | App described as facilitating family connections and support |
|  | Gamification | App described as using gamification as an intervention tool |
|  | Goal setting | App described as using goal setting or associated strategies |
|  | Havening | App explicitly described as using havening |
|  | Hypnosis | App explicitly described as using hypnosis |
|  | Lifestyle or nutrition | App described as providing information and strategies focusing on lifestyle or nutrition |
|  | Medication management | App described as facilitating management and tracking of medication |
|  | Mindfulness or meditation | App explicitly described as using mindfulness or meditation |
|  | Monitoring and tracking (including diaries) | App described as using monitoring, tracking, or diaries for recoding states, thoughts, and behaviors |
|  | Motivation enhancement | App described as using motivation enhancement or associated strategies |
|  | NLP | App explicitly described as using neuro linguistic programming |
|  | Peer support | App described as providing peer support |
|  | Positive strategies | App described as using positive strategies but does not explicitly mention use of positive psychology |
|  | Problem solving | App described as using problem solving but does not explicitly mention use of problem–solving therapy |
|  | Relaxation | App described as providing relaxation strategies and techniques |
|  | Self–compassion | App explicitly described as using self–compassion as a treatment strategy |
|  | Skills building | App described as facilitating skills building |
|  | Sounds/Music | App described as using sounds or music as an intervention |
|  | Transcranial direct current stimulation | App explicitly described as using transcranial direct current stimulation |
|  | Yoga | App explicitly described as using yoga |
| **Assessment measure** | Recorded verbatim | Name of assessment measures as listed in app store or website |
| **Disclaimer** |  |  |
|  | Yes | App store listing included medical disclaimer concerning use |
|  | No | App store listing did not include medical disclaimer concerning use |
| **Use with professional** |  |  |
|  | Yes | App store listing suggested use of the app with real–world healthcare provider |
|  | No | App store listing did not suggest use of app with real–world healthcare provider |
| **Research evidence** |  |  |
|  | Yes | Evidence of published research of app |
|  | Not published | Evidence of unpublished or internal research of app |
|  | Not found | No evidence of research of app |
