# Supplementary material for: Evaluation of Treatment Descriptions and Alignment With Clinical Guidance of Apps for Depression on App Stores: Systematic Search and Content Analysis
Source: JMIR Form Res. 2020 Nov 13;4(11):e14988. doi: 10.2196/14988 (PMC7695532; doi:10.2196/14988)
Supplement: Multimedia Appendix 3 [file formative_v4i11e14988_app3.docx]

|  |  | ACT | Assessment | BT | Complementary therapies | CBT | Cognitive training | DBT |
| --- | --- | --- | --- | --- | --- | --- | --- | --- |
| ACT | ρ | - | 0.02 | -0.01 | 0.01 | 0.27ᵃ | -0.01 | -0.01 |
|  | *P* value | - | .75 | .88 | .90 | <.001 | .83 | .81 |
| Assessment | ρ | 0.02 | - | 0.06 | -0.15 | -0.02 | 0.09 | -0.06 |
|  | *P* value | .75 | - | .26 | .004 | .65 | .11 | .28 |
| BT | ρ | -0.01 | 0.06 | - | -0.04 | 0.08 | -0.01 | -0.01 |
|  | *P* value | .88 | .26 | - | .45 | .14 | .88 | .87 |
| Complementary therapies | ρ | 0.01 | -0.15 | -0.04 | - | 0.08 | 0.01 | -0.06 |
|  | *P* value | .90 | .004 | .45 | - | .14 | .90 | .23 |
| CBT | ρ | 0.27ᵃ | -0.02 | 0.08 | 0.08 | - | 0.03 | 0.23ᵃ |
|  | *P* value | <.001 | .65 | .14 | .14 | - | .52 | <.001 |
| Cognitive training | ρ | -0.01 | 0.09 | -0.01 | 0.01 | 0.03 | - | -0.01 |
|  | *P* value | .83 | .11 | .88 | .90 | .52 | - | .81 |
| DBT | ρ | -0.01 | -0.06 | -0.01 | -0.06 | 0.23ᵃ | -0.01 | - |
|  | *P* value | .81 | .28 | .87 | .23 | <.001 | .81 | - |
| EMDR | ρ | -0.01 | -0.04 | -0.01 | 0.14ᵃ | 0.08 | -0.01 | -0.01 |
|  | *P* value | .88 | .50 | .92 | .008 | .14 | .88 | .87 |
| Exercise | ρ | -0.02 | -0.09 | -0.01 | 0.12 | 0.06 | -0.02 | 0.11 |
|  | *P* value | .71 | .09 | .79 | .02 | .26 | .71 | .04 |
| IPT | ρ | -0.01 | -0.03 | -0.004 | -0.03 | 0.13 | -0.01 | -0.01 |
|  | *P* value | .92 | .63 | .94 | .59 | .01 | .92 | .91 |
| MBCT | ρ | -0.01 | -0.03 | -0.004 | -0.03 | -0.02 | -0.01 | -0.01 |
|  | *P* value | .92 | .63 | .94 | .59 | .69 | .92 | .91 |
| MI | ρ | -0.01 | -0.03 | -0.004 | 0.10 | 0.13 | -0.01 | -0.01 |
|  | *P* value | .92 | .63 | .94 | .06 | .01 | .92 | .91 |
| Neurostimulation | ρ | -0.01 | -0.03 | -0.004 | -0.03 | -0.02 | -0.01 | -0.01 |
|  | *P* value | .92 | .63 | .94 | .59 | .69 | .92 | .91 |
| Online therapy | ρ | -0.03 | -0.08 | -0.02 | -0.13 | -0.10 | -0.03 | -0.03 |
|  | *P* value | .63 | .12 | .74 | .02 | .07 | .63 | .59 |
| Positive psychology | ρ | -0.02 | -0.09 | 0.19ᵃ | 0.08 | 0.36ᵃ | 0.12 | -0.02 |
|  | *P* value | .70 | .08 | <.001 | .16 | <.001 | .02 | .66 |
| PST | ρ | -0.01 | -0.03 | -0.004 | -0.03 | 0.13 | -0.01 | -0.01 |
|  | *P* value | .92 | .63 | .94 | .59 | .01 | .92 | .91 |
| Psychoeducation | ρ | -0.03 | -0.02 | 0.02 | -0.16 | -0.06 | 0.02 | -0.05 |
|  | *P* value | .54 | .71 | .77 | .003 | .26 | .68 | .36 |
| Psychosocial | ρ | 0.04 | -0.16 | 0.08 | -0.11 | 0.04 | -0.04 | -0.05 |
|  | *P* value | .48 | .002 | .12 | .045 | .46 | .44 | .39 |
| Faith–based | ρ | -0.01 | -0.04 | -0.01 | 0.02 | -0.04 | -0.01 | -0.01 |
|  | *P* value | .85 | .41 | .90 | .65 | .49 | .85 | .84 |
| Self–help | ρ | 0.06 | 0.07 | 0.11 | 0.06 | 0.29ᵃ | -0.03 | -0.04 |
|  | *P* value | .28 | .19 | .048 | .25 | <.001 | .52 | .47 |

Spearman rank correlation coefficient for treatment approaches ACT - DBT (N=353)

ᵃ Significant positive correlation (*P<.01)*

Spearman rank correlation coefficient for treatment approaches EMDR - Online therapy (N=353)

|  |  | EMDR | Exercise | IPT | MBCT | MI | Neurostimulation | Online therapy |
| --- | --- | --- | --- | --- | --- | --- | --- | --- |
| ACT | ρ | -0.01 | -0.02 | -0.01 | -0.01 | -0.01 | -0.01 | -0.03 |
|  | *P* value | .88 | .71 | .92 | .92 | .92 | .92 | .63 |
| Assessment | ρ | -0.04 | -0.09 | -0.03 | -0.03 | -0.03 | -0.03 | -0.08 |
|  | *P* value | .50 | .09 | .63 | .63 | .63 | .63 | .12 |
| BT | ρ | -0.01 | -0.01 | -0.004 | -0.004 | -0.004 | -0.004 | -0.02 |
|  | *P* value | .92 | .79 | .94 | .94 | .94 | .94 | .74 |
| Complementary therapies | ρ | 0.14ᵃ | 0.12 | -0.03 | -0.03 | 0.10 | -0.03 | -0.13 |
|  | *P* value | .008 | .02 | .59 | .59 | .06 | .59 | .02 |
| CBT | ρ | 0.08 | 0.06 | 0.13 | -0.02 | 0.13 | -0.02 | -0.10 |
|  | *P* value | .14 | .26 | .01 | .69 | .01 | .69 | .07 |
| Cognitive training | ρ | -0.01 | -0.02 | -0.01 | -0.01 | -0.01 | -0.01 | -0.03 |
|  | *P* value | .88 | .71 | .92 | .92 | .92 | .92 | .63 |
| DBT | ρ | -0.01 | 0.11 | -0.01 | -0.01 | -0.01 | -0.01 | -0.03 |
|  | *P* value | .87 | .04 | .91 | .91 | .91 | .91 | .59 |
| EMDR | ρ | - | 0.19ᵃ | -0.004 | -0.004 | -0.004 | -0.004 | -0.02 |
|  | *P* value | - | <.001 | .94 | .94 | .94 | .94 | .74 |
| Exercise | ρ | 0.19ᵃ | - | -0.01 | -0.01 | -0.01 | -0.01 | -0.05 |
|  | *P* value | <.001 | - | .85 | .85 | .85 | .85 | .40 |
| IPT | ρ | -0.004 | -0.01 | - | -0.003 | -0.003 | -0.003 | -0.01 |
|  | *P* value | .94 | .85 | - | .96 | .96 | .96 | .81 |
| MBCT | ρ | -0.004 | -0.01 | -0.003 | - | -0.003 | -0.003 | -0.01 |
|  | *P* value | .94 | .85 | .96 | - | .96 | .96 | .81 |
| MI | ρ | -0.004 | -0.01 | -0.003 | -0.003 | - | -0.003 | -0.01 |
|  | *P* value | .94 | .85 | .96 | .96 | - | .96 | .81 |
| Neurostimulation | ρ | -0.004 | -0.01 | -0.003 | -0.003 | -0.003 | - | -0.01 |
|  | *P* value | .94 | .85 | .96 | .96 | .96 | - | .81 |
| Online therapy | ρ | -0.02 | -0.05 | -0.01 | -0.01 | -0.01 | -0.01 | - |
|  | *P* value | .74 | .40 | .81 | .81 | .81 | .81 | - |
| Positive psychology | ρ | -0.02 | -0.04 | 0.27ᵃ | -0.01 | -0.01 | -0.01 | -0.05 |
|  | *P* value | .78 | .49 | <.001 | .85 | .85 | .85 | .38 |
| PST | ρ | -0.004 | -0.01 | 1.00ᵃ | -0.003 | -0.003 | -0.003 | -0.013 |
|  | *P* value | .94 | .85 | <.001 | .96 | .96 | .96 | .81 |
| Psychoeducation | ρ | -0.06 | -0.03 | -0.04 | 0.07 | -0.04 | -0.04 | -.17 |
|  | *P* value | .25 | .64 | .42 | .22 | .42 | .42 | 0.001 |
| Psychosocial | ρ | 0.08 | -0.03 | -0.02 | -0.02 | -0.02 | -0.02 | 0.02 |
|  | *P* value | .12 | .62 | .70 | .70 | .70 | .70 | .71 |
| Faith–based | ρ | -0.01 | -0.01 | -0.01 | -0.01 | -0.01 | -0.01 | .12 |
|  | *P* value | .90 | .75 | .93 | .93 | .93 | .93 | .03 |
| Self–help | ρ | 0.11 | -0.01 | 0.17ᵃ | -0.02 | -0.02 | -0.02 | -0.08 |
|  | *P* value | .048 | .90 | .002 | .75 | .75 | .75 | .15 |

ᵃ Significant positive correlation (*P<.01)*

Spearman rank correlation coefficient for treatment approaches Positive psychology - Self-help (N=353)

|  |  | Positive psychology | PST | Psychoeducation | Psychosocial | Faith–based | Self–help |
| --- | --- | --- | --- | --- | --- | --- | --- |
| ACT | ρ | -0.02 | -0.01 | -0.03 | 0.04 | -0.01 | 0.06 |
|  | *P* value | .70 | .92 | .54 | .48 | .85 | .28 |
| Assessment | ρ | -0.09 | -0.03 | -0.02 | -0.16 | -0.04 | 0.07 |
|  | *P* value | .08 | .63 | .71 | .002 | .41 | .19 |
| BT | ρ | 0.19ᵃ | -0.004 | 0.02 | 0.08 | -0.01 | 0.11 |
|  | *P* value | <.001 | .94 | .77 | .12 | .90 | .048 |
| Complementary therapies | ρ | 0.08 | -0.03 | -0.16 | -0.11 | 0.02 | 0.06 |
|  | *P* value | .16 | .59 | .003 | .045 | .65 | .25 |
| CBT | ρ | 0.36ᵃ | 0.13 | -0.06 | 0.04 | -0.04 | 0.29ᵃ |
|  | *P* value | <.001 | .01 | .26 | .46 | .49 | <.001 |
| Cognitive training | ρ | 0.12 | -0.01 | 0.02 | -0.04 | -0.01 | -0.03 |
|  | *P* value | .02 | .92 | .68 | .44 | .85 | .52 |
| DBT | ρ | -0.02 | -0.01 | -0.05 | -0.05 | -0.01 | -0.04 |
|  | *P* value | .66 | .91 | .36 | .39 | .84 | .47 |
| EMDR | ρ | -0.02 | -0.004 | -0.06 | 0.08 | -0.01 | 0.11 |
|  | *P* value | .78 | .94 | .25 | .12 | .90 | .048 |
| Exercise | ρ | -0.04 | -0.01 | -0.03 | -0.03 | -0.02 | -0.01 |
|  | *P* value | .49 | .85 | .64 | .62 | .75 | .90 |
| IPT | ρ | 0.27ᵃ | 1.00ᵃ | -0.04 | -0.02 | -0.01 | 0.17ᵃ |
|  | *P* value | <.001 | <.001 | .42 | .70 | .93 | .002 |
| MBCT | ρ | -0.01 | -0.003 | 0.07 | -0.02 | -0.01 | -0.02 |
|  | *P* value | .85 | .96 | .22 | .70 | .93 | .75 |
| MI | ρ | -0.01 | -0.003 | -0.04 | -0.02 | -0.01 | -0.02 |
|  | *P* value | .85 | .96 | .42 | .70 | .93 | .75 |
| Neurostimulation | ρ | -0.01 | -0.003 | -0.04 | -0.02 | -0.005 | -0.02 |
|  | *P* value | .85 | .96 | .42 | .70 | .93 | .75 |
| Online therapy | ρ | -0.05 | -0.01 | -0.17 | 0.02 | 0.12 | -0.08 |
|  | *P* value | .38 | .81 | .001 | .71 | .03 | .15 |
| Positive psychology | ρ | - | 0.27ᵃ | -0.07 | 0.06 | -0.02 | 0.14ᵃ |
|  | *P* value | - | <.001 | .21 | .27 | .76 | .007 |
| PST | ρ | 0.27ᵃ | - | -0.04 | -0.02 | -0.005 | 0.17ᵃ |
|  | *P* value | <.001 | - | .42 | .70 | .93 | .002 |
| Psychoeducation | ρ | -0.07 | -0.04 | - | -0.16 | -0.01 | 0.12 |
|  | *P* value | .21 | .42 | - | .002 | .82 | .03 |
| Psychosocial | ρ | 0.06 | -0.02 | -0.16 | - | -0.04 | 0.02 |
|  | *P* value | .27 | .70 | .002 | - | .50 | .71 |
| Faith–based | ρ | -0.02 | -0.005 | -0.01 | -0.04 | - | -0.03 |
|  | *P* value | .74 | .93 | .82 | .50 | - | .58 |
| Self–help | ρ | 0.14ᵃ^*^ | 0.17ᵃ | 0.12 | 0.02 | -0.03 | - |
|  | *P* value | .007 | .002 | .03 | .71 | .58 | - |

ᵃ Significant positive correlation (*P<.01)*
