# Supplementary material for: Evaluation of Treatment Descriptions and Alignment With Clinical Guidance of Apps for Depression on App Stores: Systematic Search and Content Analysis
Source: JMIR Form Res. 2020 Nov 13;4(11):e14988. doi: 10.2196/14988 (PMC7695532; doi:10.2196/14988)
Supplement: Multimedia Appendix 4 [file formative_v4i11e14988_app4.docx]

Spearman rank correlation coefficient for treatment strategies Acceptance - Chatbot (N=353)

|  |  | Acceptance | Acupressure | Art therapy | BA | Bodily awareness | Chatbot |
| --- | --- | --- | --- | --- | --- | --- | --- |
| Acceptance | ρ | - | -0.01 | -0.003 | -0.01 | -0.01 | -0.01 |
|  | *P* value | - | .92 | .96 | .83 | .93 | .92 |
| Acupressure | ρ | -0.01 | - | -0.01 | -0.02 | -0.01 | -0.01 |
|  | *P* value | .92 | - | .92 | .66 | .85 | .83 |
| Art therapy | ρ | -0.003 | -0.01 | - | -0.01 | -0.01 | -0.01 |
|  | *P* value | .96 | .92 | - | .83 | .93 | .92 |
| BA | ρ | -0.01 | -0.02 | -0.01 | - | -0.02 | -0.02 |
|  | *P* value | .83 | .66 | .83 | - | .71 | .66 |
| Bodily awareness | ρ | -0.01 | -0.01 | -0.01 | -0.02 | - | -0.01 |
|  | *P* value | .93 | .85 | .93 | .71 | - | .85 |
| Chatbot | ρ | -0.01 | -0.01 | -0.01 | -0.02 | -0.01 | - |
|  | *P* value | .92 | .83 | .92 | .66 | .85 | - |
| Chromotherapy | ρ | -0.003 | -0.01 | -0.003 | -0.01 | -0.01 | -0.01 |
|  | *P* value | .96 | .92 | .96 | .83 | .93 | .92 |
| Coaching | ρ | -0.01 | -0.01 | -0.01 | -0.02 | -0.01 | 0.28ᵃ |
|  | *P* value | .93 | .85 | .93 | .71 | .87 | <.001 |
| Cognitive bias modification | ρ | -0.004 | -0.01 | -0.004 | -0.02 | -0.01 | -0.01 |
|  | *P* value | .94 | .88 | .94 | .76 | .90 | .88 |
| Cognitive reappraisal | ρ | -0.02 | -0.03 | -0.02 | 0.19ᵃ | -0.03 | 0.07 |
|  | *P* value | .77 | .56 | .77 | <.001 | .62 | .19 |
| Connection to services | ρ | -0.02 | -0.03 | -0.02 | -0.01 | 0.09 | 0.07 |
|  | *P* value | .77 | .56 | .77 | .80 | .10 | .21 |
| Crisis management | ρ | -0.01 | -0.02 | -0.01 | 0.09 | 0.13 | -0.02 |
|  | *P* value | .83 | .67 | .83 | .10 | .01 | .67 |
| Distraction or grounding | ρ | -0.01 | -0.01 | -0.01 | -0.03 | -0.01 | -0.01 |
|  | *P* value | .91 | .81 | .91 | .63 | .84 | .81 |
| EFT | ρ | -0.003 | -0.01 | -0.003 | -0.01 | -0.01 | -0.01 |
|  | *P* value | .96 | .92 | .96 | .83 | .93 | .92 |
| Emotional awareness | ρ | -0.02 | -0.04 | -0.02 | 0.18ᵃ | 0.16ᵃ | 0.05 |
|  | *P* value | .72 | .47 | .72 | .001 | .003 | .40 |
| Emotion induction | ρ | -0.01 | -0.02 | -0.01 | 0.03 | 0.15ᵃ | -0.02 |
|  | *P* value | .85 | .70 | .85 | .58 | .006 | .70 |
| Exposure | ρ | -0.004 | -0.01 | -0.004 | 0.17ᵃ | -0.01 | -0.01 |
|  | *P* value | .94 | .88 | .94 | .002 | .90 | .88 |
| Family support | ρ | -0.01 | -0.02 | -0.01 | 0.03 | -0.02 | -0.02 |
|  | *P* value | .84 | .68 | .84 | .63 | .73 | .68 |
| Gamification | ρ | -0.01 | -0.01 | -0.01 | 0.28ᵃ | -0.01 | -0.01 |
|  | *P* value | .93 | .85 | .93 | <.001 | .87 | .85 |
| Goal setting | ρ | -0.01 | -0.02 | -0.01 | 0.21ᵃ | -0.02 | -0.02 |
|  | *P* value | .83 | .66 | .83 | <.001 | .71 | .66 |
| Havening | ρ | -0.003 | -0.01 | -0.003 | -0.01 | -0.01 | -0.01 |
|  | *P* value | .96 | .92 | .96 | .83 | .93 | .92 |

| Hypnosis | ρ | -0.01 | -0.02 | -0.01 | -0.05 | -0.02 | -0.02 |
| --- | --- | --- | --- | --- | --- | --- | --- |
|  | *P* value | .83 | .66 | .83 | .37 | .71 | .66 |
| Lifestyle or nutrition | ρ | -0.01 | -0.03 | -0.01 | 0.06 | -0.02 | -0.03 |
|  | *P* value | .80 | .61 | .80 | .29 | .66 | .61 |
| Medication management | ρ | -0.01 | -0.02 | -0.01 | 0.02 | -0.02 | -0.02 |
|  | *P* value | .82 | .65 | .82 | .79 | .70 | .65 |
| Mindfulness meditation | ρ | -0.02 | 0.03 | -0.02 | 0.02 | -0.04 | 0.18ᵃ |
|  | *P* value | .67 | .59 | .67 | .70 | .46 | .001 |
| Monitoring and tracking | ρ | 0.08 | -0.01 | -0.04 | 0.27ᵃ | 0.14ᵃ | 0.10 |
|  | *P* value | .14 | .80 | .51 | <.001 | .009 | .06 |
| Motivation enhancement | ρ | -0.01 | -0.01 | -0.01 | -0.02 | -0.01 | -0.01 |
|  | *P* value | .93 | .85 | .93 | .71 | .87 | .85 |
| NLP | ρ | -0.004 | -0.01 | -0.004 | -0.02 | -0.01 | -0.01 |
|  | *P* value | .94 | .88 | .94 | .76 | .90 | .88 |
| Peer support | ρ | 0.16ᵃ | -0.04 | -0.02 | -0.07 | -0.03 | -0.04 |
|  | *P* value | .002 | .51 | .75 | .18 | .57 | .51 |
| Positive strategies | ρ | -0.01 | -0.03 | -0.01 | 0.10 | 0.10 | 0.08 |
|  | *P* value | .79 | .59 | .79 | .05 | .07 | .15 |
| Problem solving | ρ | -0.01 | -0.01 | -0.01 | -0.02 | -0.01 | -0.01 |
|  | *P* value | .93 | .85 | .93 | .71 | .87 | .85 |
| Relaxation | ρ | -0.02 | 0.05 | 0.15ᵃ | 0.09 | 0.06 | 0.05 |
|  | *P* value | .72 | .40 | .006 | .10 | .24 | .40 |
| Self–compassion | ρ | -0.01 | -0.01 | -0.01 | -0.02 | -0.01 | -0.01 |
|  | *P* value | .92 | .83 | .92 | .66 | .85 | .83 |
| Skills building | ρ | -0.01 | -0.03 | -0.01 | 0.23ᵃ | -0.02 | 0.08 |
|  | *P* value | .80 | .61 | .80 | <.001 | .66 | .12 |
| Sound or music | ρ | -0.02 | 0.07 | -0.02 | -0.07 | -0.03 | -0.03 |
|  | *P* value | .77 | .22 | .77 | .22 | .60 | .55 |
| TDCS | ρ | -0.003 | -0.01 | -0.003 | -0.01 | -0.01 | -0.01 |
|  | *P* value | .96 | .92 | .96 | .83 | .93 | .92 |
| Yoga | ρ | -0.01 | -0.02 | -0.01 | 0.05 | -0.02 | 0.14ᵃ |
|  | *P* value | .87 | .73 | .87 | .40 | .77 | .007 |

ᵃ Significant positive correlation (*P<.01)*

Spearman rank correlation coefficient for treatment strategies Chromotherapy - Crisis management (N=353)

|  |  | Chromotherapy | Coaching | Cognitive bias modification | Cognitive reappraisal | Connection to services | Crisis management |
| --- | --- | --- | --- | --- | --- | --- | --- |
| Acceptance | ρ | -0.003 | -0.01 | -0.004 | -0.02 | -0.02 | -0.01 |
|  | *P* value | .96 | .93 | .94 | .77 | .77 | .83 |
| Acupressure | ρ | -0.01 | -0.01 | -0.01 | -0.03 | -0.03 | -0.02 |
|  | *P* value | .92 | .85 | .88 | .56 | .56 | .67 |
| Art therapy | ρ | -0.003 | -0.01 | -0.004 | -0.02 | -0.02 | -0.01 |
|  | *P* value | .96 | .93 | .94 | .77 | .77 | .83 |
| BA | ρ | -0.01 | -0.02 | -0.02 | 0.19ᵃ | -0.01 | 0.09 |
|  | *P* value | .83 | .71 | .76 | <.001 | .80 | .10 |
| Bodily awareness | ρ | -0.01 | -0.01 | -0.01 | -0.03 | 0.09 | 0.13 |
|  | *P* value | .93 | .87 | .90 | .62 | .10 | .01 |
| Chatbot | ρ | -0.01 | 0.28ᵃ | -0.01 | 0.07 | 0.07 | -0.02 |
|  | *P* value | .92 | <.001 | .88 | .19 | .21 | .67 |
| Chromotherapy | ρ | - | -0.01 | -0.004 | -0.02 | -0.02 | -0.01 |
|  | *P* value | - | .93 | .94 | .77 | .77 | .83 |
| Coaching | ρ | -0.01 | - | -0.01 | 0.09 | -0.03 | 0.13 |
|  | *P* value | .93 | - | .90 | .09 | .61 | .01 |
| Cognitive bias modification | ρ | -0.004 | -0.01 | - | -0.02 | -0.02 | -0.02 |
|  | *P* value | .94 | .90 | - | .68 | .68 | .77 |
| Cognitive reappraisal | ρ | -0.02 | 0.09 | -0.02 | - | -0.08 | 0.05 |
|  | *P* value | .77 | .09 | .68 | - | .11 | .40 |
| Connection to services | ρ | -0.02 | -0.03 | -0.02 | -0.08 | - | 0.30ᵃ |
|  | *P* value | .77 | .61 | .68 | .11 | - | <.001 |
| Crisis management | ρ | -0.01 | 0.13 | -0.02 | 0.05 | 0.30ᵃ | - |
|  | *P* value | .83 | .01 | .77 | .40 | <.001 | - |
| Distraction or grounding | ρ | -0.01 | 0.25ᵃ | -0.01 | 0.15ᵃ | 0.05 | 0.21ᵃ |
|  | *P* value | .91 | <.001 | .87 | .006 | .32 | <.001 |
| EFT | ρ | -0.003 | -0.01 | -0.004 | -0.02 | -0.02 | -0.01 |
|  | *P* value | .96 | .93 | .94 | .77 | .77 | .83 |
| Emotional awareness | ρ | -0.02 | -0.03 | -0.03 | 0.30ᵃ | -0.04 | -0.08 |
|  | *P* value | .72 | .53 | .61 | <.001 | .44 | .15 |
| Emotion induction | ρ | -0.01 | -0.02 | 0.19ᵃ | -0.06 | 0.05 | 0.11 |
|  | *P* value | .85 | .74 | <.001 | .29 | .31 | .04 |
| Exposure | ρ | -0.004 | -0.01 | -0.01 | 0.12 | -0.02 | -0.02 |
|  | *P* value | .94 | .90 | .92 | .02 | .68 | .77 |
| Family support | ρ | -0.01 | -0.02 | -0.02 | -0.004 | 0.21ᵃ | 0.17ᵃ |
|  | *P* value | .84 | .73 | .77 | .94 | <.001 | .001 |
| Gamification | ρ | -0.01 | -0.01 | -0.01 | -0.03 | -0.03 | 0.13 |
|  | *P* value | .93 | .87 | .90 | .62 | .61 | .01 |
| Goal setting | ρ | -0.01 | 0.13 | -0.02 | 0.30ᵃ | -0.06 | -0.05 |
|  | *P* value | .83 | .02 | .76 | <.001 | .23 | .39 |
| Havening | ρ | -0.003 | -0.01 | -0.004 | -0.02 | -0.02 | -0.01 |
|  | *P* value | .96 | .93 | .94 | .77 | .77 | .83 |

| Hypnosis | ρ | -0.01 | -0.02 | -0.02 | -0.06 | -0.06 | -0.05 |
| --- | --- | --- | --- | --- | --- | --- | --- |
|  | *P* value | .83 | .71 | .76 | .24 | .23 | .39 |
| Lifestyle or nutrition | ρ | -0.01 | -0.02 | -0.02 | 0.01 | -0.08 | 0.004 |
|  | *P* value | .80 | .66 | .72 | .79 | .16 | .94 |
| Medication management | ρ | -0.01 | -0.02 | -0.02 | -0.02 | 0.03 | 0.15ᵃ |
|  | *P* value | .82 | .70 | .75 | .78 | .55 | .005 |
| Mindfulness meditation | ρ | -0.02 | 0.13 | 0.07 | 0.12 | -0.13 | -0.01 |
|  | *P* value | .67 | .01 | .17 | .03 | .02 | .83 |
| Monitoring and tracking | ρ | -0.04 | 0.07 | 0.03 | 0.36ᵃ | 0.03 | 0.13 |
|  | *P* value | .51 | .18 | .56 | <.001 | .57 | .01 |
| Motivation enhancement | ρ | -0.01 | -0.01 | -0.01 | -0.03 | -0.03 | 0.13 |
|  | *P* value | .93 | .87 | .90 | .62 | .61 | .01 |
| NLP | ρ | -0.004 | -0.01 | -0.01 | -0.02 | -0.02 | -0.02 |
|  | *P* value | .94 | .90 | .92 | .68 | .68 | .77 |
| Peer support | ρ | -0.02 | -0.03 | -0.03 | -0.02 | 0.08 | -0.07 |
|  | *P* value | .75 | .57 | .64 | .69 | .13 | .20 |
| Positive strategies | ρ | -0.01 | 0.10 | -0.02 | 0.05 | -0.04 | -0.001 |
|  | *P* value | .79 | .07 | .70 | .36 | .48 | .98 |
| Problem solving | ρ | -0.01 | -0.01 | -0.01 | 0.09 | -0.03 | -0.02 |
|  | *P* value | .93 | .87 | .90 | .09 | .61 | .72 |
| Relaxation | ρ | 0.15ᵃ | 0.16ᵃ | 0.21ᵃ | 0.13 | -0.04 | 0.10 |
|  | *P* value | .006 | .003 | <.001 | .02 | .44 | .06 |
| Self–compassion | ρ | -0.01 | -0.01 | -0.01 | 0.07 | -0.03 | -0.02 |
|  | *P* value | .92 | .85 | .88 | .19 | .56 | .67 |
| Skills building | ρ | -0.01 | 0.23ᵃ | -0.02 | 0.32ᵃ | 0.01 | 0.24ᵃ |
|  | *P* value | .80 | <.001 | .72 | <.001 | .84 | <.001 |
| Sound or music | ρ | -0.02 | -0.03 | -0.02 | -0.09 | -0.05 | -0.06 |
|  | *P* value | .77 | .60 | .67 | .11 | .35 | .24 |
| TDCS | ρ | -0.003 | -0.01 | -0.004 | -0.02 | -0.02 | -0.01 |
|  | *P* value | .96 | .93 | .94 | .77 | .77 | .83 |
| Yoga | ρ | -0.01 | 0.17ᵃ | -0.01 | -0.05 | -0.05 | -0.04 |
|  | *P* value | .87 | .001 | .81 | .36 | .35 | .50 |

ᵃ Significant positive correlation (*P<.01)*

Spearman rank correlation coefficient for treatment strategies Distancing/Grounding - Family support (N=353)

|  |  | Distraction or  grounding | EFT | Emotional awareness | Emotion induction | Exposure | Family support |
| --- | --- | --- | --- | --- | --- | --- | --- |
| Acceptance | ρ | -0.01 | -0.003 | -0.02 | -0.01 | -0.004 | -0.01 |
|  | *P* value | .91 | .96 | .72 | .85 | .94 | .84 |
| Acupressure | ρ | -0.01 | -0.01 | -0.04 | -0.02 | -0.01 | -0.02 |
|  | *P* value | .81 | .92 | .47 | .70 | .88 | .68 |
| Art therapy | ρ | -0.01 | -0.003 | -0.02 | -0.01 | -0.004 | -0.01 |
|  | *P* value | .91 | .96 | .72 | .85 | .94 | .84 |
| BA | ρ | -0.03 | -0.01 | 0.18ᵃ | 0.03 | 0.17ᵃ | 0.03 |
|  | *P* value | .63 | .83 | .001 | .58 | .002 | .63 |
| Bodily awareness | ρ | -0.01 | -0.01 | 0.16ᵃ | 0.15ᵃ | -0.01 | -0.02 |
|  | *P* value | .84 | .93 | .003 | .006 | .90 | .73 |
| Chatbot | ρ | -0.01 | -0.01 | 0.05 | -0.02 | -0.01 | -0.02 |
|  | *P* value | .81 | .92 | .40 | .70 | .88 | .68 |
| Chromotherapy | ρ | -0.01 | -0.003 | -0.02 | -0.01 | -0.004 | -0.01 |
|  | *P* value | .91 | .96 | .72 | .85 | .94 | .84 |
| Coaching | ρ | 0.25ᵃ | -0.01 | -0.03 | -0.02 | -0.01 | -0.02 |
|  | *P* value | <.001 | . 93 | .53 | .74 | .90 | .73 |
| Cognitive bias modification | ρ | -0.01 | -0.004 | -0.03 | 0.19ᵃ | -0.01 | -0.02 |
|  | *P* value | .87 | .94 | .61 | <.001 | .92 | .77 |
| Cognitive reappraisal | ρ | 0.15ᵃ | -0.02 | 0.30ᵃ | -0.06 | 0.12 | -0.004 |
|  | *P* value | .006 | .77 | <.001 | .29 | .02 | .94 |
| Connection to services | ρ | 0.05 | -0.02 | -0.04 | 0.05 | -0.02 | 0.21ᵃ |
|  | *P* value | .32 | .77 | .44 | .31 | .68 | <.001 |
| Crisis management | ρ | 0.21ᵃ | -0.01 | -0.08 | 0.11 | -0.02 | 0.17ᵃ |
|  | *P* value | <.001 | .83 | .15 | .04 | .77 | .001 |
| Distraction or grounding | ρ | - | -0.01 | -0.04 | 0.23ᵃ | -0.01 | -0.02 |
|  | *P* value | - | .91 | .42 | <.001 | .87 | .65 |
| EFT | ρ | -0.01 | - | 0.15ᵃ | -0.01 | 0.71ᵃ | -0.01 |
|  | *P* value | .91 | - | .006 | .85 | <.001 | .84 |
| Emotional awareness | ρ | -0.04 | 0.15ᵃ | - | 0.02 | 0.21ᵃ | -0.03 |
|  | *P* value | .42 | .006 | - | .67 | <.001 | .60 |
| Emotion induction | ρ | 0.23ᵃ | -0.01 | 0.02 | - | -0.02 | -0.04 |
|  | *P* value | <.001 | .85 | .67 | - | .78 | .46 |
| Exposure | ρ | -0.01 | 0.71ᵃ | 0.21ᵃ | -0.02 | - | -0.02 |
|  | *P* value | .87 | <.001 | <.001 | .78 | - | .77 |
| Family support | ρ | -0.02 | -0.01 | -0.03 | -0.04 | -0.02 | - |
|  | *P* value | .65 | .84 | .60 | .46 | .77 | - |
| Gamification | ρ | -0.01 | -0.01 | -0.03 | -0.02 | -0.01 | 0.14ᵃ |
|  | *P* value | .84 | .93 | .53 | .74 | .90 | .009 |
| Goal setting | ρ | 0.09 | -0.01 | 0.30ᵃ | 0.10 | -0.02 | 0.03 |
|  | *P* value | .10 | .83 | <.001 | .06 | .76 | .63 |
| Havening | ρ | -0.01 | 1.00ᵃ | 0.15ᵃ | -0.01 | 0.71ᵃ | -0.01 |
|  | *P* value | .91 | <.001 | .006 | .85 | <.001 | .84 |

| Hypnosis | ρ | -0.03 | 0.25ᵃ | -0.04 | -0.04 | 0.17ᵃ | -0.04 |
| --- | --- | --- | --- | --- | --- | --- | --- |
|  | *P* value | .63 | <.001 | .49 | .43 | .002 | .41 |
| Lifestyle or nutrition | ρ | -0.03 | -0.01 | -0.02 | 0.01 | -0.02 | -0.05 |
|  | *P* value | .56 | .80 | .71 | .83 | .72 | .33 |
| Medication management | ρ | -0.03 | -0.01 | 0.08 | 0.03 | -0.02 | 0.16ᵃ |
|  | *P* value | .61 | .82 | .12 | .62 | .75 | .003 |
| Mindfulness meditation | ρ | 0.02 | 0.13 | 0.04 | 0.00 | 0.07 | -0.05 |
|  | *P* value | .77 | .02 | .43 | .99 | .17 | .39 |
| Monitoring and tracking | ρ | 0.13 | 0.08 | 0.52ᵃ | 0.13 | 0.03 | 0.18ᵃ |
|  | *P* value | .02 | .14 | <.001 | .02 | .56 | .001 |
| Motivation enhancement | ρ | -0.01 | -0.01 | -0.03 | -0.02 | -0.01 | -0.02 |
|  | *P* value | .84 | .93 | .53 | .74 | .90 | .73 |
| NLP | ρ | -0.01 | 0.71ᵃ | 0.09 | -0.02 | 0.50ᵃ | -0.02 |
|  | *P* value | .87 | <.001 | .09 | .78 | <.001 | .77 |
| Peer support | ρ | 0.04 | 0.16ᵃ | 0.06 | -0.01 | 0.10 | 0.03 |
|  | *P* value | .43 | .002 | .25 | .81 | .05 | .55 |
| Positive strategies | ρ | -0.03 | 0.20ᵃ | 0.18ᵃ | 0.13 | 0.13 | 0.003 |
|  | *P* value | .54 | <.001 | .001 | .02 | .02 | .96 |
| Problem solving | ρ | 0.25ᵃ | 0.58ᵃ | 0.06 | -0.02 | 0.40ᵃ | -0.02 |
|  | *P* value | <.001 | <.001 | .24 | .74 | <.001 | .73 |
| Relaxation | ρ | 0.03 | 0.15ᵃ | 0.06 | 0.21ᵃ | 0.09 | -0.03 |
|  | *P* value | .56 | .006 | .25 | <.001 | .09 | .60 |
| Self–compassion | ρ | -0.01 | 0.50ᵃ | 0.21ᵃ | -0.02 | 0.35 | -0.02 |
|  | *P* value | .81 | <.001 | <.001 | .70 | <.001 | .68 |
| Skills building | ρ | 0.27ᵃ | 0.21ᵃ | 0.13 | 0.01 | 0.29ᵃ | 0.01 |
|  | *P* value | <.001 | <.001 | .02 | .83 | <.001 | .89 |
| Sound or music | ρ | -0.04 | 0.18ᵃ | -0.08 | -0.004 | 0.12 | -0.06 |
|  | *P* value | .50 | .001 | .15 | .94 | .03 | .26 |
| TDCS | ρ | -0.01 | -0.003 | -0.02 | -0.01 | -0.004 | -0.01 |
|  | *P* value | .91 | .96 | .72 | .85 | .94 | .84 |
| Yoga | ρ | -0.02 | 0.31ᵃ | -0.01 | 0.06 | 0.22ᵃ | -0.04 |
|  | *P* value | .70 | <.001 | .87 | .28 | <.001 | .52 |

ᵃ Significant positive correlation (*P<.01)*

Spearman rank correlation coefficient for treatment strategies Gamification - Medication management (N=353)

|  |  | Gamification | Goal setting | Havening | Hypnosis | Lifestyle or  nutrition | Medication management |
| --- | --- | --- | --- | --- | --- | --- | --- |
| Acceptance | ρ | -0.01 | -0.01 | -0.003 | -0.01 | -0.01 | -0.01 |
|  | *P* value | .93 | .83 | .96 | .83 | .80 | .82 |
| Acupressure | ρ | -0.01 | -0.02 | -0.01 | -0.02 | -0.03 | -0.02 |
|  | *P* value | .85 | .66 | .92 | .66 | .61 | .65 |
| Art therapy | ρ | -0.01 | -0.01 | -0.003 | -0.01 | -0.01 | -0.01 |
|  | *P* value | .93 | .83 | .96 | .83 | .80 | .82 |
| BA | ρ | 0.28ᵃ | 0.21ᵃ | -0.01 | -0.05 | 0.06 | 0.02 |
|  | *P* value | <.001 | <.001 | .83 | .37 | .29 | .79 |
| Bodily awareness | ρ | -0.01 | -0.02 | -0.01 | -0.02 | -0.02 | -0.02 |
|  | *P* value | .87 | .71 | .93 | .71 | .66 | .70 |
| Chatbot | ρ | -0.01 | -0.02 | -0.01 | -0.02 | -0.03 | -0.02 |
|  | *P* value | .85 | .66 | .92 | .66 | .61 | .65 |
| Chromotherapy | ρ | -0.01 | -0.01 | -0.003 | -0.01 | -0.01 | -0.01 |
|  | *P* value | .93 | .83 | .96 | .83 | .80 | .82 |
| Coaching | ρ | -0.01 | 0.13 | -0.01 | -0.02 | -0.02 | -0.02 |
|  | *P* value | .87 | .02 | .93 | .71 | .66 | .70 |
| Cognitive bias modification | ρ | -0.01 | -0.02 | -0.004 | -0.02 | -0.02 | -0.02 |
|  | *P* value | .90 | .76 | .94 | .76 | .72 | .75 |
| Cognitive reappraisal | ρ | -0.03 | 0.30ᵃ | -0.02 | -0.06 | 0.01 | -0.02 |
|  | *P* value | .62 | <.001 | .77 | .24 | .79 | .78 |
| Connection to services | ρ | -0.03 | -0.06 | -0.02 | -0.06 | -0.08 | 0.03 |
|  | *P* value | .61 | .23 | .77 | .23 | .16 | .55 |
| Crisis management | ρ | 0.13 | -0.05 | -0.01 | -0.05 | 0.004 | 0.15ᵃ |
|  | *P* value | .01 | .39 | .83 | .39 | .94 | .005 |
| Distraction or grounding | ρ | -0.01 | 0.09 | -0.01 | -0.03 | -0.03 | -0.03 |
|  | *P* value | .84 | .10 | .91 | .63 | .56 | .61 |
| EFT | ρ | -0.01 | -0.01 | 1.00ᵃ | 0.25ᵃ | -0.01 | -0.01 |
|  | *P* value | 0.926 | 0.828 | <.001 | <.001 | .80 | .82 |
| Emotional awareness | ρ | -0.03 | 0.30ᵃ | 0.15 | -0.04 | -0.02 | 0.08 |
|  | *P* value | .53 | <.001 | .01 | .49 | .70 | .12 |
| Emotion induction | ρ | -0.02 | 0.10 | -0.01 | -0.04 | 0.01 | 0.03 |
|  | *P* value | .74 | .06 | .85 | .43 | .83 | .63 |
| Exposure | ρ | -0.01 | -0.02 | 0.71ᵃ | 0.17ᵃ | -0.02 | -0.02 |
|  | *P* value | .90 | .76 | <.001 | .002 | .72 | .75 |
| Family support | ρ | 0.14ᵃ | 0.03 | -0.01 | -0.04 | -0.05 | 0.16ᵃ |
|  | *P* value | .009 | .63 | .84 | .41 | .33 | .003 |
| Gamification | ρ | - | -0.02 | -0.01 | -0.02 | -0.02 | 0.12 |
|  | *P* value | - | .71 | .93 | .71 | .66 | .02 |
| Goal setting | ρ | -0.02 | - | -0.01 | 0.02 | 0.00 | -0.05 |
|  | *P* value | .71 | - | .83 | .74 | .99 | .36 |
| Havening | ρ | -0.01 | -0.01 | - | 0.25ᵃ | -0.01 | -0.01 |
|  | *P* value | .93 | .83 | - | <.001 | .80 | .82 |

| Hypnosis | ρ | -0.02 | 0.02 | 0.25ᵃ | - | -0.06 | -0.05 |
| --- | --- | --- | --- | --- | --- | --- | --- |
|  | *P* value | .71 | .74 | <.001 | - | .29 | .36 |
| Lifestyle or nutrition | ρ | -0.02 | 0.00 | -0.01 | -0.06 | - | -0.06 |
|  | *P* value | .66 | .99 | .80 | .29 | - | .28 |
| Medication management | ρ | 0.12 | -0.05 | -0.01 | -0.05 | -0.06 | - |
|  | *P* value | .02 | .36 | .82 | .36 | .28 | - |
| Mindfulness meditation | ρ | -0.04 | 0.13 | 0.13 | 0.06 | -0.04 | -0.10 |
|  | *P* value | .46 | .01 | .02 | .27 | .41 | .07 |
| Monitoring and tracking | ρ | 0.07 | 0.27ᵃ | 0.08 | -0.12 | -0.05 | 0.34ᵃ |
|  | *P* value | .18 | <.001 | .14 | .03 | .39 | <.001 |
| Motivation enhancement | ρ | -0.01 | -0.02 | -0.01 | -0.02 | -0.02 | -0.02 |
|  | *P* value | .87 | .71 | .93 | .71 | .66 | .70 |
| NLP | ρ | -0.01 | 0.17ᵃ | 0.71ᵃ | 0.35ᵃ | -0.02 | -0.02 |
|  | *P* value | .90 | .002 | <.001 | <.001 | .72 | .75 |
| Peer support | ρ | -0.03 | 0.02 | 0.16ᵃ | -0.03 | -0.08 | 0.06 |
|  | *P* value | .57 | .69 | .002 | .64 | .11 | .25 |
| Positive strategies | ρ | -0.03 | 0.32ᵃ | 0.20ᵃ | -0.01 | -0.07 | -0.06 |
|  | *P* value | .64 | <.001 | <.001 | .93 | .19 | .26 |
| Problem solving | ρ | -0.01 | -0.02 | 0.58ᵃ | 0.13 | -0.02 | -0.02 |
|  | *P* value | .87 | .71 | <.001 | .02 | .66 | .70 |
| Relaxation | ρ | 0.06 | 0.22ᵃ | 0.15ᵃ | 0.01 | -0.02 | -0.04 |
|  | *P* value | .24 | <.001 | .006 | .91 | .70 | .45 |
| Self–compassion | ρ | -0.01 | -0.02 | 0.50ᵃ | 0.11 | -0.03 | -0.02 |
|  | *P* value | .85 | .66 | <.001 | .05 | .61 | .65 |
| Skills building | ρ | 0.23ᵃ | 0.17ᵃ | 0.21ᵃ | 0.00 | -0.02 | 0.05 |
|  | *P* value | <.001 | .001 | <.001 | .99 | .74 | .34 |
| Sound or music | ρ | -0.03 | -0.07 | 0.18ᵃ | 0.43ᵃ | -0.03 | -0.07 |
|  | *P* value | .60 | .22 | .001 | <.001 | .52 | .21 |
| TDCS | ρ | -0.01 | -0.01 | -0.003 | -0.01 | -0.01 | -0.01 |
|  | *P* value | .93 | .83 | .96 | .83 | .80 | .82 |
| Yoga | ρ | -0.02 | -0.04 | 0.31ᵃ | 0.05 | 0.03 | -0.04 |
|  | *P* value | .77 | .49 | <.001 | .40 | .62 | .47 |

ᵃ Significant positive correlation (*P<.01)*

Spearman rank correlation coefficient for treatment strategies Mindfulness/Meditation – Positive strategies (N=353)

|  |  | Mindfulness meditation | Monitoring and tracking | Motivation enhancement | NLP | Peer support | Positive strategies |
| --- | --- | --- | --- | --- | --- | --- | --- |
| Acceptance | ρ | -0.02 | 0.08 | -0.01 | -0.004 | 0.16ᵃ | -0.01 |
|  | *P* value | .67 | .14 | .93 | .94 | .002 | .79 |
| Acupressure | ρ | 0.03 | -0.01 | -0.01 | -0.01 | -0.04 | -0.03 |
|  | *P* value | .59 | .80 | .85 | .88 | .51 | .59 |
| Art therapy | ρ | -0.02 | -0.04 | -0.01 | -0.004 | -0.02 | -0.01 |
|  | *P* value | .67 | .51 | .93 | .94 | .75 | .79 |
| BA | ρ | 0.02 | 0.27ᵃ | -0.02 | -0.02 | -0.07 | 0.10 |
|  | *P* value | .70 | <.001 | .71 | .76 | .18 | .05 |
| Bodily awareness | ρ | -0.04 | 0.14ᵃ | -0.01 | -0.01 | -0.03 | 0.10 |
|  | *P* value | .46 | .009 | .87 | .90 | .57 | .07 |
| Chatbot | ρ | 0.18ᵃ | 0.10 | -0.01 | -0.01 | -0.04 | 0.08 |
|  | *P* value | .001 | .06 | .85 | .88 | .51 | .15 |
| Chromotherapy | ρ | -0.02 | -0.04 | -0.01 | -0.004 | -0.02 | -0.01 |
|  | *P* value | .67 | .51 | .93 | .94 | .75 | .79 |
| Coaching | ρ | 0.13 | 0.07 | -0.01 | -0.01 | -0.03 | 0.10 |
|  | *P* value | .01 | .18 | .87 | .90 | .57 | .07 |
| Cognitive bias modification | ρ | 0.07 | 0.03 | -0.01 | -0.01 | -0.03 | -0.02 |
|  | *P* value | .17 | .56 | .90 | .92 | .64 | .70 |
| Cognitive reappraisal | ρ | 0.12 | 0.36ᵃ | -0.03 | -0.02 | -0.02 | 0.05 |
|  | *P* value | .03 | <.001 | .62 | .68 | .69 | .36 |
| Connection to services | ρ | -0.13 | 0.03 | -0.03 | -0.02 | 0.08 | -0.04 |
|  | *P* value | .02 | .57 | .61 | .68 | .13 | .48 |
| Crisis management | ρ | -0.01 | 0.13 | 0.13 | -0.02 | -0.07 | -0.001 |
|  | *P* value | .83 | .01 | .01 | .77 | .20 | .98 |
| Distraction and grounding | ρ | 0.02 | 0.13 | -0.01 | -0.01 | 0.04 | -0.03 |
|  | *P* value | .77 | .02 | .84 | .87 | .43 | .54 |
| EFT | ρ | 0.13 | 0.08 | -0.01 | 0.71ᵃ | 0.16ᵃ | 0.20ᵃ |
|  | *P* value | .02 | .14 | .93 | <.001 | .002 | <.001 |
| Emotional awareness | ρ | 0.04 | 0.52ᵃ | -0.03 | 0.09 | 0.06 | 0.18ᵃ |
|  | *P* value | .43 | <.001 | .53 | .09 | .25 | .001 |
| Emotion induction | ρ | 0.00 | 0.13 | -0.02 | -0.02 | -0.01 | 0.13 |
|  | *P* value | .99 | .02 | .74 | .78 | .81 | .02 |
| Exposure | ρ | 0.07 | 0.03 | -0.01 | 0.50ᵃ | 0.10 | 0.13 |
|  | *P* value | .17 | .56 | .90 | <.001 | .05 | .02 |
| Family support | ρ | -0.05 | 0.18ᵃ | -0.02 | -0.02 | 0.03 | 0.003 |
|  | *P* value | .39 | .001 | .73 | .77 | .55 | .96 |
| Gamification | ρ | -0.04 | 0.07 | -0.01 | -0.01 | -0.03 | -0.03 |
|  | *P* value | .46 | .18 | .87 | .90 | .57 | .64 |
| Goal setting | ρ | 0.13 | 0.27ᵃ | -0.02 | 0.17ᵃ | 0.02 | 0.32ᵃ |
|  | *P* value | .01 | <.001 | .71 | .002 | .69 | <.001 |
| Havening | ρ | 0.13 | 0.08 | -0.01 | 0.71ᵃ | 0.16ᵃ | 0.20ᵃ |
|  | *P* value | .02 | .12 | .93 | <.001 | .002 | <.001 |

| Hypnosis | ρ | 0.06 | -0.12 | -0.02 | 0.35ᵃ | -0.03 | -0.01 |
| --- | --- | --- | --- | --- | --- | --- | --- |
|  | *P* value | .27 | .03 | .71 | <.001 | .64 | .93 |
| Lifestyle or nutrition | ρ | -0.04 | -0.05 | -0.02 | -0.02 | -0.08 | -0.07 |
|  | *P* value | .41 | .39 | .66 | .72 | .11 | .19 |
| Medication management | ρ | -0.10 | 0.34ᵃ | -0.02 | -0.02 | 0.06 | -0.06 |
|  | *P* value | .07 | <.001 | .70 | .75 | .25 | .26 |
| Mindfulness meditation | ρ | - | 0.06 | 0.05 | 0.07 | -0.03 | 0.20ᵃ |
|  | *P* value | - | .29 | .39 | .17 | .55 | <.001 |
| Monitoring and tracking | ρ | 0.06 | - | 0.01 | 0.03 | 0.05 | 0.09 |
|  | *P* value | .29 | - | .93 | .56 | .33 | .10 |
| Motivation enhancement | ρ | 0.05 | 0.01 | - | -0.01 | -0.03 | 0.10 |
|  | *P* value | .39 | .93 | - | .90 | .57 | .07 |
| NLP | ρ | 0.07 | 0.03 | -0.01 | - | 0.10 | 0.13 |
|  | *P* value | .17 | .56 | .90 | - | .05 | .02 |
| Peer support | ρ | -0.03 | 0.05 | -0.03 | 0.10 | - | 0.03 |
|  | *P* value | .55 | .33 | .57 | .05 | - | .62 |
| Positive strategies | ρ | 0.20ᵃ | 0.09 | 0.10 | 0.13 | 0.03 | - |
|  | *P* value | <.001 | .10 | .07 | .02 | .62 | - |
| Problem solving | ρ | 0.05 | 0.07 | -0.01 | 0.40ᵃ | 0.07 | 0.22ᵃ |
|  | *P* value | .39 | .18 | .87 | <.001 | .16 | <.001 |
| Relaxation | ρ | 0.39ᵃ | 0.14ᵃ | 0.06 | 0.09 | 0.002 | 0.22ᵃ |
|  | *P* value | <.001 | .008 | .24 | .09 | 0.98 | <.001 |
| Self–compassion | ρ | 0.10 | 0.10 | -0.01 | 0.35ᵃ | 0.06 | 0.18ᵃ |
|  | *P* value | .05 | .06 | .85 | <.001 | .30 | .001 |
| Skills building | ρ | 0.22 | 0.21ᵃ | 0.10 | 0.14 | -0.04 | 0.26ᵃ |
|  | *P* value | <.001 | <.001 | .05 | .01 | .41 | <.001 |
| Sound or music | ρ | 0.13 | -0.11 | -0.03 | 0.12 | -0.06 | 0.04 |
|  | *P* value | .01 | .04 | .60 | .03 | .24 | .43 |
| TDCS | ρ | -0.02 | 0.08 | -0.01 | -0.004 | -0.02 | -0.01 |
|  | *P* value | .67 | .14 | .93 | .94 | .75 | .79 |
| Yoga | ρ | 0.26ᵃ | -0.08 | -0.02 | 0.22ᵃ | 0.002 | 0.23ᵃ |
|  | *P* value | <.001 | .15 | .77 | <.001 | .97 | <.001 |

ᵃ Significant positive correlation (*P<.01)*

Spearman rank correlation coefficient for treatment strategies Problem solving - Yoga (N=353)

|  |  | Problem solving | Relaxation | Self-compassion | Skills building | Sound or music | TDCS | Yoga |
| --- | --- | --- | --- | --- | --- | --- | --- | --- |
| Acceptance | ρ | -0.01 | -0.02 | -0.01 | -0.01 | -0.02 | -0.003 | -0.01 |
|  | *P* value | .93 | .72 | .92 | .80 | .77 | .96 | .87 |
| Acupressure | ρ | -0.01 | 0.05 | -0.01 | -0.03 | 0.07 | -0.01 | -0.02 |
|  | *P* value | .85 | .40 | .83 | .61 | .22 | .92 | .73 |
| Art therapy | ρ | -0.01 | 0.15ᵃ | -0.01 | -0.01 | -0.02 | -0.003 | -0.01 |
|  | *P* value | .93 | .006 | .92 | .80 | .77 | .96 | .87 |
| BA | ρ | -0.02 | 0.09 | -0.02 | 0.23ᵃ | -0.07 | -0.01 | 0.05 |
|  | *P* value | .71 | .09 | .66 | <.001 | .22 | .83 | .40 |
| Bodily awareness | ρ | -0.01 | 0.06 | -0.01 | -0.02 | -0.03 | -0.01 | -0.02 |
|  | *P* value | .87 | .24 | .85 | .66 | .60 | .93 | .77 |
| Chatbot | ρ | -0.01 | 0.05 | -0.01 | 0.08 | -0.03 | -0.01 | 0.14ᵃ |
|  | *P* value | .85 | .40 | .83 | .12 | .55 | .92 | .007 |
| Chromotherapy | ρ | -0.01 | 0.15ᵃ | -0.01 | -0.01 | -0.02 | -0.003 | -0.01 |
|  | *P* value | .93 | .006 | .92 | .80 | .77 | .96 | .87 |
| Coaching | ρ | -0.01 | 0.16ᵃ | -0.01 | 0.23ᵃ | -0.03 | -0.01 | 0.17ᵃ |
|  | *P* value | .87 | .003 | .85 | <.001 | .60 | .93 | .001 |
| Cognitive bias modification | ρ | -0.01 | 0.21ᵃ | -0.01 | -0.02 | -0.02 | -0.004 | -0.01 |
|  | *P* value | .90 | <.001 | .88 | .72 | .67 | .94 | .81 |
| Cognitive reappraisal | ρ | 0.09 | 0.13 | 0.07 | 0.32ᵃ | -0.09 | -0.02 | -0.05 |
|  | *P* value | .09 | .02 | .19 | <.001 | .11 | .77 | .36 |
| Connection to services | ρ | -0.03 | -0.04 | -0.03 | 0.01 | -0.05 | -0.02 | -0.05 |
|  | *P* value | .61 | .44 | .56 | .84 | .35 | .77 | .35 |
| Crisis management | ρ | -0.02 | 0.10 | -0.02 | 0.24ᵃ | -0.06 | -0.01 | -0.04 |
|  | *P* value | .72 | .06 | .67 | <.001 | .24 | .83 | .50 |
| Distraction or grounding | ρ | 0.25ᵃ | 0.03 | -0.01 | 0.27ᵃ | -0.04 | -0.01 | -0.02 |
|  | *P* value | <.001 | .56 | .81 | <.001 | .50 | .91 | .70 |
| EFT | ρ | 0.58ᵃ | 0.15ᵃ | 0.50ᵃ | 0.21ᵃ | 0.18ᵃ | -0.003 | 0.31ᵃ |
|  | *P* value | <.001 | .006 | <.001 | <.001 | .001 | .96 | <.001 |
| Emotional awareness | ρ | 0.06 | 0.06 | 0.21ᵃ | 0.13 | -0.08 | -0.02 | -0.01 |
|  | *P* value | .24 | .25 | <.001 | .02 | .15 | .72 | .87 |
| Emotion induction | ρ | -0.02 | 0.21ᵃ | -0.02 | 0.01 | -0.004 | -0.01 | 0.06 |
|  | *P* value | .74 | <.001 | .70 | .83 | .94 | .85 | .28 |
| Exposure | ρ | 0.40ᵃ | 0.09 | 0.35ᵃ | 0.29ᵃ | 0.12 | -0.004 | 0.22ᵃ |
|  | *P* value | <.001 | .09 | <.001 | <.001 | .03 | .94 | <.001 |
| Family support | ρ | -0.02 | -0.03 | -0.02 | 0.01 | -0.06 | -0.01 | -0.04 |
|  | *P* value | .73 | .60 | .68 | .89 | .26 | .84 | .52 |
| Gamification | ρ | -0.01 | 0.06 | -0.01 | 0.23ᵃ | -0.03 | -0.01 | -0.02 |
|  | *P* value | .87 | .24 | .85 | <.001 | .60 | .93 | .77 |
| Goal setting | ρ | -0.02 | 0.22ᵃ | -0.02 | 0.17ᵃ | -0.07 | -0.01 | -0.04 |
|  | *P* value | .71 | <.001 | .66 | .001 | .22 | .83 | .49 |
| Havening | ρ | .58ᵃ | 0.15ᵃ | 0.50ᵃ | 0.21ᵃ | 0.18ᵃ | -0.003 | 0.31ᵃ |
|  | *P* value | <.001 | .006 | <.001 | <.001 | .001 | .96 | <.001 |

| Hypnosis | ρ | 0.13 | 0.01 | 0.11 | 0.00 | 0.43ᵃ | -0.01 | 0.05 |
| --- | --- | --- | --- | --- | --- | --- | --- | --- |
|  | *P* value | .02 | .91 | .05 | .99 | <.001 | .83 | .40 |
| Lifestyle or nutrition | ρ | -0.02 | -0.02 | -0.03 | -0.02 | -0.03 | -0.01 | 0.03 |
|  | *P* value | .66 | .70 | .61 | .74 | .52 | .80 | .62 |
| Medication management | ρ | -0.02 | -0.04 | -0.02 | 0.05 | -0.07 | -0.01 | -0.04 |
|  | *P* value | .70 | .45 | .65 | .34 | .21 | .82 | .47 |
| Mindfulness meditation | ρ | 0.05 | 0.39ᵃ | 0.10 | 0.22ᵃ | 0.13 | -0.02 | 0.26ᵃ |
|  | *P* value | .39 | <.001 | .05 | <.001 | .01 | .67 | <.001 |
| Monitoring and tracking | ρ | 0.07 | 0.14ᵃ | 0.10 | 0.21ᵃ | -0.11 | 0.08 | -0.08 |
|  | *P* value | .18 | .008 | .06 | <.001 | .04 | .14 | .15 |
| Motivation enhancement | ρ | -0.01 | 0.06 | -0.01 | 0.10 | -0.03 | -0.01 | -0.02 |
|  | *P* value | .87 | .24 | .85 | .05 | .60 | .93 | .77 |
| NLP | ρ | 0.40ᵃ | 0.09 | 0.35ᵃ | 0.14 | 0.12 | -0.004 | 0.22ᵃ |
|  | *P* value | <.001 | .09 | <.001 | .01 | .03 | .94 | <.001 |
| Peer support | ρ | 0.07 | 0.002 | 0.06 | -0.04 | -0.06 | -0.02 | 0.002 |
|  | *P* value | .16 | .98 | .30 | .41 | .24 | .75 | .97 |
| Positive strategies | ρ | 0.22ᵃ | 0.22ᵃ | 0.18ᵃ | 0.26ᵃ | 0.04 | -0.01 | 0.23ᵃ |
|  | *P* value | <.001 | <.001 | .001 | <.001 | .43 | .79 | <.001 |
| Problem solving | ρ | - | 0.06 | 0.28ᵃ | 0.23ᵃ | 0.09 | -0.01 | 0.17ᵃ |
|  | *P* value | - | .24 | <.001 | <.001 | .11 | .93 | .001 |
| Relaxation | ρ | 0.06 | - | 0.05 | 0.20ᵃ | 0.09 | -0.02 | 0.26ᵃ |
|  | *P* value | .24 | - | .40 | <.001 | .11 | .72 | <.001 |
| Self–compassion | ρ | 0.28ᵃ | 0.05 | - | 0.19ᵃ | 0.07 | -0.01 | 0.14ᵃ |
|  | *P* value | <.001 | .40 | - | <.001 | .22 | .92 | .007 |
| Skills building | ρ | 0.23ᵃ | 0.20ᵃ | 0.19ᵃ | - | -0.03 | -0.01 | 0.10 |
|  | *P* value | <.001 | <.001 | <0.001 | - | .52 | .80 | .07 |
| Sound or music | ρ | 0.09 | 0.09 | 0.07 | -0.03 | - | -0.02 | 0.14 |
|  | *P* value | .11 | .11 | .22 | .52 | - | .77 | .01 |
| TDCS | ρ | -0.01 | -0.02 | -0.01 | -0.01 | -0.02 | - | -0.01 |
|  | *P* value | .93 | .72 | .92 | .80 | .77 | - | .87 |
| Yoga | ρ | 0.17ᵃ | 0.26ᵃ | 0.14ᵃ | 0.10 | 0.14 | -0.01 | - |
|  | *P* value | .001 | <.001 | .007 | .07 | .01 | .87 | - |

ᵃ Significant positive correlation (*P<.01)*
